# Spatial and temporal variations of geochemical processes and toxicity of water, sediments, and suspended solids in Sibuti River Estuary, NW Borneo

**DOI:** 10.1007/s11356-023-28596-5

**Published:** 2023-07-26

**Authors:** Rakesh Roshan Gantayat, Prasanna Mohan Viswanathan, Nagarajan Ramasamy, Chidambaram Sabarathinam

**Affiliations:** 1 Department of Applied Sciences, Faculty of Engineering and Science, Curtin University Malaysia, CDT 250, 98009 Miri, Sarawak Malaysia; 2 Department of Hydrology, University of Zululand, Kwadlangezwa, South Africa; 3 Water Research Center, Kuwait Institute for Scientific Research, Safat, Kuwait

**Keywords:** Geochemistry, Ion exchange, Redox, Absorption, Metal mobility, Sibuti River, Estuary

## Abstract

A comprehensive geochemical study was conducted in the Sibuti River estuary by considering water, suspended solids (SS), and sediment samples from 36 stations during southwest monsoon (SWM) and northeast monsoon (NEM). In this study, the distribution of in situ parameters, major ions, nutrients, trace metals, and isotopes (δD, δ^18^O) were analyzed in water samples, whereas sediments and SS were studied for trace metals. The distribution revealed that suspended solids were the major carrier of Cd, Zn, and Mn, whereas sediments worked as a major source of Co, Cr, Ba, Se, Cu, and Pb. Na-Cl water type and ion exchange dominated the lower part of the estuary during both seasons. However, the mixed mechanism of Ca–Cl, Ca–Mg–Cl, and higher weathering indicated reverse ion exchange in the intermediate and upper parts of the estuary. Isotopic signatures of δD and δ^18^O in estuarine water indicate that the precipitation over the Limbang area dominates during SWM, whereas higher evaporation was confirmed during NEM. The factor analysis revealed that seawater influence in the estuary majority controlled the water chemistry irrespective of seasons. Major ions were mainly regulated by the tidal influence during the low flow time of the river (SWM), whereas the mixing mechanism of weathering and seawater controlled the concentrations during NEM. Nutrients such as NO_3_, SO_4_^2−^, NH_3_, and NH_4_^+^ mainly originated from the agricultural fields and nitrification along with ammonification were responsible for the recycling of such nutrients. Trace metals except Cd were found to be geogenic in nature and originating mainly from the oxidation of pyrites present in the sandstone and mudstones of the Sibuti Formation. Redox condition was catalyzed by microorganisms near the river mouth, whereas Al-oxyhydroxides and Fe-oxyhydroxides complexes in the intermediate and upper part under oxygenated conditions controlled the absorption of metals. Overall, the estuary was found to be absorptive in nature due to ideal pH conditions and was confirmed by the saturation index (SI) of minerals.

**Supplementary Information:**

The online version contains supplementary material available at 10.1007/s11356-023-28596-5.

## Introduction

Globally, rivers are the essential participants of geochemical, biochemical, and elemental cycles due to their nature of being a primary facilitator in the transport of terrestrial inputs through sediments, suspended and in dissolved form (Reiman et al. 2018). The key drivers of such changes in a river’s ecosystem are always the inputs by the headwaters, in its catchment area or floodplains. On the other hand, estuaries are the transitional zone of the river where the mixing of two water masses (seawater and river water) of distinct physico-chemical properties takes place while working as a geochemical filter for contaminants carried in form of sediment (suspended and bed load) and dissolved load (Guinoiseau et al. 2016; Koukina et al. 2021). The origin of these contaminants generally attributes to anthropogenic sources such as agriculture and urbanized run-off (Somura et al. 2012; Priya et al. 2014; Frazar et al. 2019; Asha et al. 2020; Huang et al. 2020) and natural sources such as weathering of rocks in the basinal region (Pavoni et al. 2021), which contributes the majority of the solutes and solid matters to the river system (Martin and Whitfield 1983; Lintern et al. 2018).

The primary functions of the river is mainly controlled by the three interfaces such as water, suspended particles, and sediment load. Sediments are considered as the most important interface to be paid attention to as the global contribution of river-derived sediments comprises up to 95% of the material entering the world’s oceans (Lučić et al. 2019). In addition to that, 30 to 98% of toxic materials like metals from geogenic/anthropogenic sources transported by rivers get deposited on sediments under varying environmental conditions (Gibbs 1973; Yang and Wang 2017). The study of sediment composition in estuaries helps to reflect the geochemical nature of the river basin and helps to elucidate the overall character of the material transported by rivers from adjacent land areas, derived from shoreline erosion, carried by marine currents from external sources, and produced in situ by organisms and contribution by human activities (Prabakaran et al. 2020). On the other hand, rivers around the world carry 13.5 × 10^9^ tons of suspended solids towards the sea (Eisma 1988), and estuaries work as geochemical and biochemical reactors that modify the river fluxes and composition of terrestrial inputs within its influencing territory (Sholkovitz and Szymczak 2000; Koukina et al. 2021). Such processes are mainly controlled by physical and chemical factors such as hydrodynamic mixing due to volatile tidal gradient (Salas-Monreal and Valle-Levinson 2008; Mathew and Winterwerp 2020) whereas fluvial effect and geomorphological processes coupled with rock-water interaction contribute the majority of weathered inputs and are highly dependent on seasonal rainfall (Ralston et al. 2013; Brantley et al. 2017; Mathew & Winterwerp 2020). In such an environment, cohesive aggregates play a major role in the sorption of various suspended and dissolved chemical constituents (Mehta 1989; Kronvang et al. 2003). The deviating nature of these particles from bedload sediments allows them to mobilize frequently and follow much shorter cycles than the resistant sediment particles at the bottom (Eisma 1988). The availability in the water table besides working as a medium between metals that are dissolved and in the solid phase, a much larger surface area, and the presence of clay minerals and organic matter which are far more surface-active than comparatively larger sediments allow them to absorb/desorb higher concentration of metals (Regnier and Wollast 1993).

Furthermore, the formation of turbidity maxima zones (TMZ) with enhanced sediment tapping zones (Ishak et al. 2001; Mathew and Winterwerp 2020) and complex chemical reactions such as dissolution/precipitation (Juen et al. 2015; Naderi et al. 2016; Vinh and Ouillon 2021), ion exchange (Patra et al. 2012; Cochran 2014; Hao et al. 2020), water density stratification leading oxidation–reduction process (e.g., Ishak et al. 2001; Walker et al. 2021; Tian 2020), flocculation (e.g., Karbassi et al. 2016; Zhang et al. 2020), and absorption/desorption (e.g., Hirst et al. 2017; Mohamed and Yaacob 2019; Zhou et al. 2020) are the major mechanisms behind metal transition between particulate and dissolved medium. In a similar manner, the tidal influence and seasonal rainfall also control the residence time of water in estuaries giving rise to nutrient cycles such as N-cycle and P-cycle (e.g., Zhu et al. 2018; Geisler et al. 2020; Wei et al. 2022) along with higher dissolution/precipitation of minerals in the estuarine system depending upon the CO_2_ balance with atmosphere (Prasanna et al. 2010; Chidambaram et al. 2011; Naderi et al. 2016). The highly dynamic nature of estuaries with volatile tidal and geomorphological gradients provide a unique environment for such complex trace metal and nutrient cycling (Turner 1996).

The distribution of trace metals and nutrients in solid, liquid, and colloidal or particulate phases plays a major role in determining the water quality and health of estuarine and coastal ecosystems (Tomczak et al. 2019; Iglesias et al. 2020; Koukina et al. 2021). As a result, these inputs work as a foundation for the survival of aquatic life while assisting in the primary production of nutrients such as N and P and the structural development of organisms; they also can aggravate adverse impacts on coastal and estuarine biota along with human health when present in overwhelming quantity (Jezierska et al. 2009; Tchounwou et al. 2012; Reiman et al. 2018). In the case of tropical river basins, intense rainfall coupled with warm and humid climate leads to intense weathering and transportation of terrestrial materials towards coastal regions in a shorter period compared to temperate climates (Koukina et al. 2021). Therefore, the study of estuaries of such rivers are ideal to study the geochemistry of water, sediment, and suspended solids (SS).

One of such estuaries is the Sibuti River estuary in Sarawak, Borneo, which discharges directly into the South China Sea (SCS). The previous studies conducted in the river estuary have been very limited to nutrients and some physico-chemical parameters (Gandaseca et al. 2011; Saifullah et al. 2014). The quantification and distribution of major ions, nutrients, and trace metals in water, sediments, and suspended solids and their governing geochemical processes have been lacking and remain a major gap. Consequently, this study is aimed at (1) filling the gap with the help of a geochemical baseline study of water, sediment, and suspended solid phase and (2) gaining an understanding of chemical processes controlling these ions, nutrients, and trace metals in these three different phases. To achieve the objectives of this study, a multivariate approach has been implemented, which includes (1) implementation of geochemical plots (e.g., Piper and Gibbs plots) for water and statistical techniques such as factor analysis and principal component analysis (PCA) for water, sediments, and suspended solids to study geochemistry; (2) thermodynamic stability between the solid and dissolved phase was analyzed using saturation index (SI) for water, whereas partition coefficient (*K*_d_) coupled with Pearson’s correlation was utilized to understand the metal transition between both phases, and (3) stable isotopes such as δD and δ^18^O were integrated with the study to identify the source of precipitation and origin of water in the estuary.

## Study area

The Sibuti River estuary is situated in the Miri zone in the north-western part of Sarawak, which is one of the states of Malaysia that covers part of Borneo Island. The river has a catchment area of 1020 km^2^ (Tenaga 2003) and discharges into South China Sea. The river catchment receives an average seasonal rainfall similar to the regional rainfall (3126 to 3246 mm, average: 3022 mm) and spanning over 220 days a year. The rainfall in the study area follows a similar pattern as regional and is mainly regulated by PDO (Pacific Decadal Oscillation) as mentioned before bringing two monsoon seasons in a year such as southwest monsoon (SWM) and northeast monsoon (NEM). This monsoonal effect is mainly responsible for the hydrological balance in this region and for controlling the river run-offs (Krawczyk et al. 2020; Naciri et al. 2023; Browne et al. 2019). Similarly, the NE monsoon is associated with higher rainfall than the SW monsoon. The river receives tides at a maximum height of 2–3 m and falls under tide-dominated estuary, especially in micro-tidal type (tidal height: 2–3 m) (Boothroyd 1978), where limited tidal influence can be expected irrespective of the season. The estuary has more than 30 km of pristine mangrove and Nipa palm (Mangrove palm) forest in the vicinity with several agricultural fields, small towns such as Bekenu, and several villages that are present around the river. The major land use activity in the river basin is agriculture and is mainly concentrated around Bekenu and several other villages. Bekenu’s growing agro-activity drives in this region are a major source of income for the locals. The main agricultural products of these areas might include palm tree plantation, pandan coconut plantation, lemon grass, ginger, turmeric, shallots, chilies, and other herbs (MANRED, Sarawak 2020). The river is also accompanied by several tributaries such as Sungai Tiris and Sungai Kejapil along its path and mainly drains the sedimentary terrains and agricultural lands along the way. The current study focuses mainly on the estuarine region of the river starting from the river mouth to Balau village.

### Lithology

The river basin is mostly influenced by three major and six minor formations, namely, the Sibuti Formation, Lambir Formation, and alluvium near the coast, whereas the minor formations may include Miri, Belait, Tukau, Nyalau, Setap shale, and Tangap formations). The basin is mainly represented by sedimentary rocks ranging from Oligocene to Pliocene. Sibuti and Lambir formations have a prominent presence in the river basin and cover 500.43 and 238.201 km^2^ of area, respectively. Both the formations are formed by the recycling, transportation, and deposition of sediments from the collision zone of the Rajang group (Nagarajan et al. 2015, 2017a) caused by the event termed as Sarawak orogeny (Hutchison 1996; Hutchison 2005). The Sibuti Formation is reported to be dominated by calcareous mudstone/shale and sandstone (Nagarajan et al. 2017a) with prominent marl lenses, thin limestone beds, and high content of fossils in it (Peng et al. 2004; Nagarajan et al. 2015). The siliciclastic sediments of this formation are rich in light minerals such as quartz, mica, calcite, minor feldspar and zeolites, and clay minerals (i.e., illite, chlority, and kaolinite), along with heavy minerals such as zircon, rutile, pyrite, and ilmenite (Nagarajan et al. 2015, 2019). The Lambir Formation is mainly comprised of sandstones, sandy intercalations with shale and siltstones, mudstone, and limestone. Mineralogically, sandstones are comprised of quartz, illite/muscovite, along with a minor amount of plagioclase, whereas limestone consists of calcite, ankerite, quartz, chlorite, illite/muscovite, and a trace amount of aragonite (Nagarajan et al. 2017a). In addition, mudstones of this formation are dominated by quartz, illite/muscovite, amorphous phase, chlorite, plagioclase, and calcite (Nagarajan et al. 2017a, 2019). Apart from these major lithological details, the concretion of pyrites was reported to be present in Sibuti Formation (Azrul NIsyam et al. 2013; Nagarajan et al. 2019) and Tukau Formation (Nagarajan et al. 2017b, 2022), and these formations are also part of the Sibuti River basin.

## Materials and methods

### Sample collection and preparation

**Fig. 1:**
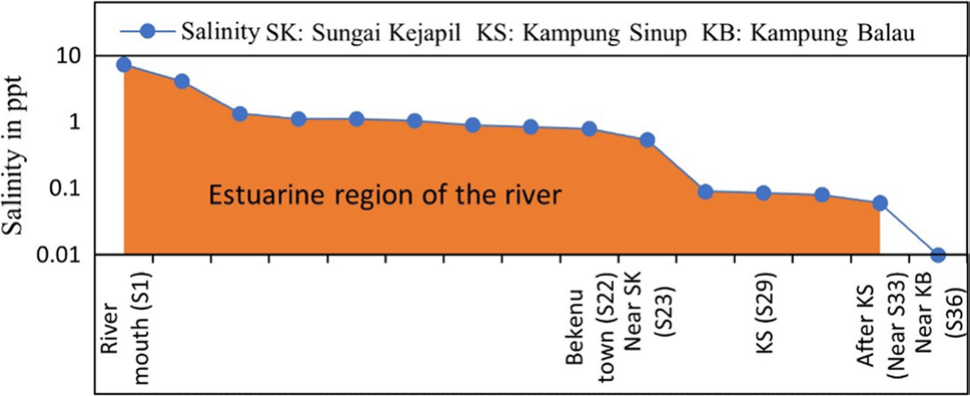
Determined estuarine boundary during preliminary studies

To assess monsoonal inputs, their associated parameters, and changes in the estuarine system, the sampling processes were planned and executed during SWM and NEM periods. The tidal water in estuaries is the major short-term influencer of spatial and chemical changes due to the intrusion of saline water from the sea and is helpful in demarcating the boundary of estuaries. Thus, high tidal conditions and salinity were taken into consideration during the demarcation of the estuarine boundary (Fig. 1). During the sampling process, observed tidal conditions were noted to assess their influence on riverine constituents during both seasons.

**Fig. 2:**
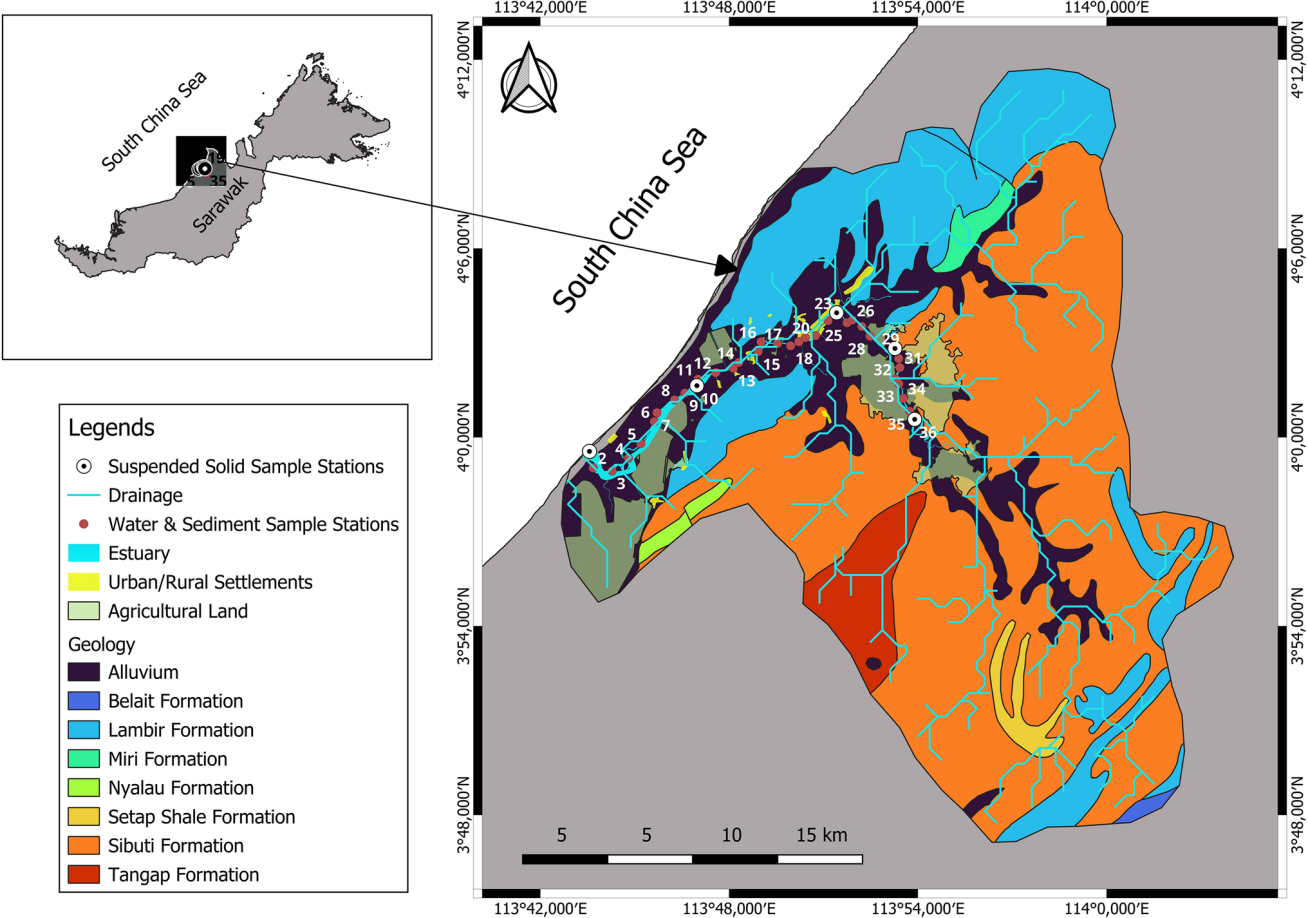
Map of Sibuti river basin showing sampling stations, geological formations and land use pattern

Water, bed load sediment, and suspended solid samples were collected in August 2017 (SW monsoon) and February 2018 (NE monsoon). High and low tidal conditions were observed during SWM on consecutive days whereas high-to-low tidal transition was observed on consecutive days during NEM. Sampling sites were selected depending upon some important aspects, such as the influence of tributaries, evidence of anthropogenic activities such as settlements and agricultural channels and meanders/major turnings. Thirty-six sites were confirmed, and 3 L of water samples were collected from each station in 3 clean polyethylene sample containers with depth reaching a maximum of 1–2 m at every station (Fig. 2). All 3 L of water were filtered with 0.45-µm Whatman filter paper, and total suspended solid (TSS) amount collected per liter of water through filtration was recorded for each station. The first bottle of water was used for nutrient (SO_4_^2−^, PO_4_^3−^, NH_3_, NH_4_^+^, NO_3_^−^, and NO_3_-N) analysis. The second bottle of water was used for the measurement of various major ion concentrations in water such as Cl^−^, CO_3_^−^, HCO_3_^−^, Ca^2+^, and Mg^2+^, whereas the third bottle of water was acidified to pH < 2 for the determination of trace metals and major ions such as Na^+^ and K^+^ concentration in water using nitric acid (30%). These samples were stored in a refrigerator at 4 °C until further processes such as digestion and trace metal analysis. Similarly, 1 kg of bed load sediments was collected from the same 36 stations using an Ekman grab sampler and stored in a plastic container and sealed to avoid any contamination. The central portion of the grab samples was considered to avoid contamination from the wall of the sampler. The sampler was washed on the river water before and after the sampling at each location and followed throughout the sampling. The sediment samples were collected in the middle of the river at each station. In addition to 1 L water samples collected from each stations to quantify the total suspended solids (TSS), ~ 20 L of water samples were collected at an interval of at least 10 km (Fig. 2) to obtain enough suspended solids for the bulk geochemical analysis. The interval has been considered to identify the changes occurring in the suspended solids amount and its constituents with respect to the river, its tributaries, and influence of tides in relation to the resuspension of the sediments along the estuary and various geochemical processes.

### Field measurements

Physico-chemical parameters such as pH, temperature, electrical conductivity (EC), total dissolved solids (TDS), salinity, turbidity, and dissolved oxygen (DO) were measured in situ in the field. Among the parameters, pH, temperature, EC, TDS, and DO were measured using respective probes in Lovibond meter. The turbidity of the water was measured with a turbidity meter, and a Hach salinity probe and meter were used to measure the salinity. Apart from this, a flow meter (Valeport current flow meter) was used to measure the velocity of water at all the sampling locations. The depth of velocity measurement was kept at 3–4 m (maximum reach of flow meter). All the probes and meters were calibrated before the fieldwork commenced at the laboratory.

### Sample analysis

The water samples collected from 72 stations (36 samples per season) were digested using acid digestion method 3005A (USEPA 1992), where HNO_3_ and HCl were used as the main reagents for digestion. This digestion was done for the analysis of ions such as Na^+^, K^+^, and trace metals such as Co, Cu, Mn, Pb, Zn, Se, Fe, Al, Cd, Cr, and Ba in water. The analysis of these ions and metals was carried out using Flame Atomic Absorption Spectroscopy (Perkin Elmar A Analyst 400). The concentration of major ions (Cl^−^, CO_3_^−^, HCO_3_^−^, Ca^2+^, and Mg^2+^) and CO_2_ was done using the titrimetric method (APHA 1998), whereas nutrients (SO_4_^2−^, PO_4_^3−^, NH_3_, NH_4_^+^, NO_3_^−^, and NO_3_-N) were analyzed in Hach DR-2800 portable Spectrophotometer using Hack test kits such as NO_3_^−^ (cadmium reduction method), NH_3_-N (salicylate method), PO_4_^3−^ (ascorbic acid method), and SO_4_^2−^ (sulfaVer 4 method).

Sediment samples were brought to the laboratory and dried in the oven at 60 °C. The dried samples were homogenized using a stone mortar and pestle. Meanwhile, the roots, leaves, gravels, and other anthropogenic/natural vegetable matter were removed manually. The samples were sieved for particle size analysis. The sediments collected in the pan with < 63 microns were used to perform the digestion by the Perkin Elmer Titan MPS Microwave digestion system. So, the present study reports the bulk geochemistry of the fine fraction of the sediments (< 63 µm) and their mechanisms. In the case of SS, collected 20 L water samples were stored for 2 weeks to settle the suspended solids down. Once they were settled, the upper half of the water layer was pumped out from the container using a small submersible pump without disturbing the lower half of the tank and filtration through a 0.45-µm filter paper was done using a vacuum pump. The water in the lower half of the container was subjected to a centrifuge to extract the settled suspended solids. The samples were dried at 60 °C to remove the water content and subjected to total digestion using Perkin Elmar Titan MPS Microwave Digester.

Two hundred milligrams of both the sediments and SS samples were digested using Perkins Elmar Titan MPS predefined method (Perkin 2013) with 2 mL of hydrofluoric acid (HF; 49%) and 6.6 mL of hydrochloric acid (HCl; 39%). After the digestion, 2 mL of boric acid (H_3_BO_3_) was added into the digested solution and heated using a hotplate to reduce the complexity of HF in the solution. The analysis for metals (Al, Cr, Mn, Fe, Co, Cu, Zn, Cd, Ba, Se, and Pb) was done using Flame Atomic Absorption Spectroscopy (Perkin Elmar A Analyst 400).

### Calibration and data accuracy

For the accuracy of Perkin Elmar A Analyst 400, calibration curves were obtained first using the standards prepared from the stock and sub-stock. These standard curves were obtained before the analysis of water and sediment samples. The instrument conditions and detection limits are given in the supplementary Table 1. One preliminary analysis was also done prior to the analysis to check the limits of concentration of metals in the samples and the range was adjusted according to the need depending on the obtained results. The correlation coefficient (*R*^2^) for each metal was determined from the calibration curve and made sure that it was above 0.995 to ensure reliable results. To ensure stability in the accuracy of results, quality check (QC) was done at every 20-sample interval throughout the analysis. Standard reference materials such as MESS-3 and BCR-701 were utilized for quality control during AAS analysis.

### Data analysis

#### Geochemistry and statistical analysis

To understand the geochemical processes, variability and distribution of different parameter factor analysis were employed using SPSS software (version 20) for the water, floor sediments, and suspended solids separately by adapting principal component analysis (PCA) with varimax rotation. The correlated variables which were linearly related form one factor represented as a gradient (eigen vector) in multidimensional space. Varimax rotation was utilized for the analysis, where each factor is independent of the others and those with eigenvalues > 1 were considered for interpretation. In addition, PCA was utilized to transform original factor data into a form that can be evaluated in multidimensional (Euclidean) space. Additionally, to identify the water types, base exchange, and chemistry, Piper plot (Piper 1944), IBE (Schoeller 1967) and Gibbs plot (Gibbs 1970) were utilized.

#### Saturation index

The thermodynamic stability of estuarine water with respect to specific mineral compositions was estimated using WATEQ4F and its integrated database. Such modeling helps to identify mineral and gas mole transfers that account for differences in the composition of an initial and final water within specified compositional uncertainty limits (Ledesma-Ruiz et al. 2015). The SI can be calculated by the log-ratio value of ionic activity product in water (IAP) (Ferrer et al. 1988) with respect to the solubility product of the mineral (Ksp).

1 $$\mathrm{SI}=\mathrm{log}\left(\mathrm{IAP}/{\mathrm{K}}_{\mathrm{sp}}\right)$$

#### Isotopic analysis

Oxygen (δ^18^O) and deuterium (δD) isotopes were analyzed at Isotope Hydrology Division of the Center for Water Resource Development and Management (CWRDM), India, using continuous-flow isotope ratio mass spectrometry (FINNIGAN DELTA^PLUS^ XP). International isotope standards (VSMOW and GFLES-1) were used during periodical calibration of the instruments. Stable isotope values were represented by *δ* (expressed in terms of parts per million, ‰) and defined as

2 $$\delta(\permille)=(\frac{R_{\mathrm{sample}}-R_{\mathrm{std}}}{R_{\mathrm{std}}})\times10^3$$

where *R* is D/H or ^18^O/^16^O and SMOW is the Standard Mean Ocean Water. A regression line between δ^18^O and δD was derived from the water samples collected around the world and referred to as Global Meteoric Water Line (GMWL) (Craig 1961). This GMWL is expressed as

3 $$\delta\mathrm{D}\;(\permille)=8\delta^{18}\mathrm O+10$$

The d-excess (excess of deuterium), which explains the connection of water to the kinetic fractionation of falling raindrops and the vapor source of regional meteorological conditions can be obtained using the equation given below (Dansgaard 1964)

4 $$\mathrm d-\mathrm{excess}\;(\permille)=\;\delta\mathrm{D}-8\delta^{18}\mathrm O$$

#### Partitioning of metals between particulate and dissolved phase

The partitioning of trace metals in the aquatic system can be impacted by various controlling parameters such as pH, salinity, turbidity, and amount of SS in the water column (Zhang et al. 2018; Yang and Wang 2017; Kumar et al. 2010). In such a scenario, the calculation of the partition coefficient (*K*_d_) helps to evaluate the partitioning balance of trace metals between the particulate phase and liquid phase (Zheng et al. 2013; Zhang et al. 2018). The calculation was done for trace metals such as Co, Cu, Mn, Zn, Se, Fe, Cd, Ba, and Cr depending on their concentration in the particulate phase (Me_p_) and metals/metalloids in the dissolved phase (Me_d_), where Me_p_ is considered in mg kg^−1^ and Me_d_ is considered in mg L^−1^.

5 $${K}_{\mathrm{d}}={~}^{{\mathrm{Me}}_{\mathrm{p}}}\!\left/ \!{~}_{{\mathrm{Me}}_{\mathrm{d}}}\right.$$

*K*_d_ is represented by the distribution co-efficient and expressed in mg metal per kg. The higher value of *K*_d_ (> 3) shows the affinity of metals towards SS or absorption whereas, a lower value than 3 represents a higher affinity of metals towards liquid phase or dissolution under varying environmental conditions (Kumar et al. 2010; Zheng et al. 2013; Zhang et al. 2018; Yang and Wang 2017; Sedeño-Díaz et al. 2020).

## Results and discussion

### Hydrochemistry

The descriptive statistics of the parameters measured in water, sediment and SS during SWM and NEM is presented in Table 1, and the actual elemental concentrations are given in supplementary Table 2. The spatial distribution of physico-chemical parameters is given in supplementary Fig. 1. Salinity in the study area near the river mouth has the highest values during both SWM and NEM mainly due to tidal water influence, especially at station 1 (nearest to the sea) (supplementary Fig. 2). Parameters such as EC and TDS followed a similar trend as salinity. TSS and turbidity were higher and gradually increased towards the lower reaches of the estuary during SWM, whereas these parameters found higher in the upper reaches during NEM. DO was found lesser in the lower part during SWM whereas found higher during NEM in the same region, which may be due to the higher infusion of freshwater due to high rainfall. High pH was recorded in the upper part of the estuary as compared to the lower part for both seasons. A frequent fluctuation of velocity was observed in the estuary during both seasons, and higher velocity was recorded in the lower part, which might be governed by an oscillatory gradient of tidal water during SWM due to high tide or steady gradients in subtidal water level during NEM due to low tide (Sassi and Hoitink 2013). In case of major ions, concentration of Cl^−^ and Na^+^ are found highest in concentration during both seasons. Overall mean abundance of ions during SWM and NEM can be observed as Cl^−^  > Na^+^  > SO_4_^2−^  > HCO_3_^−^  > K^+^  > Ca^2+^  > Mg^2+^ and Cl^−^  > Na^+^  > Mg^2+^  > Ca^2+^  > SO_4_^2−^  > HCO_3_^−^  > K^+^, respectively. Among the measured nutrients, PO_4_^3−^ and NO_3_ dominated the estuary during SWM and NEM. The mean abundance of nutrients can be observed as PO_4_^3−^  > NH_3_ > NH_4_^+^  > NO_3_^−^  > NO_3_-N in SWM and NO_3_^−^  > NH_4_^+^  > NH_3_ > NO_3_-N > PO_4_^3−^ during NEM. The dominancy of PO_4_^3−^ is contributed by the saline sediments presence in the estuary and low flow of the river where terrigenous Fe (III) bounds to P deposited in the sediment while releasing PO_4_^3−^ into the water column (Hartzell and Jordan 2012), whereas prevailing freshwater conditions during NEM have reduced the abundance of PO_4_^3−^ in estuarine water. The concentrations of all metals during NEM were higher than SWM except for Cr, Mn, Ba, and Se. The concentrations of Se and Fe have significant dominance in the estuarine waters during both seasons. The mean dominance of metals in water can be observed as Se > Fe > Cr > Mn > Zn > Ba > Cu > Cd > Co during SWM and Fe > Se > Cu > Co > Zn > Cr > Zn > Ba > Cd = Pb during NEM. Similarly, Fe was found to be the dominating metal in suspended solids for both seasons, and a higher concentration of Fe is noticed during SWM, whereas vice versa condition prevailed in the case of Mn in the estuary. The absence of dissolved Al in water during both seasons and the high concentration of Al in SS indicates that it is originating from catchment areas as detrital input. In sediments, Fe and Al were the dominating metals irrespective of the seasons. The average values of both metals indicates higher concentration during SWM as compared to NEM (Table 1). The abundance of considered metals during SWM and NEM are as follows Al > Fe > Ba > Co > Cu > Se > Mn > Zn > Cr > Pb > Cd and Fe > Al > Co > Ba > Se > Cu > Mn > Zn > Cr > Cd > Pb.

**Table 1:** Descriptive statistics of elemental concentrations in water, suspended solids, and sediments, including isotopes in water

Parameters	Units	SWM				NEM			
		Min	Max	Mean	St. Dev	Min	Max	Mean	St. Dev
Physico-chemical parameters									
pH	-	4.6	6.88	6.16	0.33	5.45	7.6	6.61	0.4
DO	mg L^−1^	3.66	6.44	4.44	0.44	13.2	35.1	20.49	5.15
EC	µS cm^−1^	107.6	3170	409.6	719.63	139.59	11,971.44	1707.25	2805.01
TDS	mg L^−1^	153.8	4528.6	585.15	1028.05	97.71	8380.01	1195.08	1963.51
Turbidity	NTU	7.87	53.9	27.9	9.61	15.1	50.1	31.69	8.37
Velocity	m/s	0.7	11.84	3.58	2.02	0.29	2	0.88	0.48
Salinity	ppt	0.02	1.65	0.61	0.59	0.04	11.7	1.05	2.12
Temp	°C	21.4	30	28.4	1.39	29.8	32	30.04	0.44
CO_2_	mg L^−1^	4.4	15.4	6.91	2.42	11	39.6	20.96	5.84
TSS	mg L^−1^	20.2	178.6	55.62	39.3	44	384.3	156.81	92.33
Major ion concentrations in water									
Ca^2+^	mg L^−1^	2	14	4.83	3.1	2	286	60.58	67.59
Mg^2+^	mg L^−1^	BDL	54	4.7	9.47	BDL	1082.4	125.92	233.3
Na^+^	mg L^−1^	20.4	1252.8	134.7	261.2	2.76	3034	292.97	582.29
K^+^	mg L^−1^	7.96	75.05	15.89	13.1	2.88	98.26	17.1	16.3
HCO_3_^−^	mg L^−1^	18.3	54.9	31.35	7.46	12.2	61	27.45	10
Cl^−^	mg L^−1^	26.58	2153.58	182.17	441.71	17.72	5742.9	621.85	1131.22
SO_4_^2−^	mg L^−1^	6	89	34.97	20.35	2	133	46.86	45.05
Nutrient concentrations in water									
PO_4_^3−^	mg L^−1^	BDL	3.45	0.87	0.92	0.03	0.35	0.11	0.06
NO_3_-N	mg L^−1^	BDL	0.08	0.03	0.02	BDL	0.86	0.19	0.32
NH_3_	mg L^−1^	0.01	0.9	0.18	0.23	0.17	0.97	0.58	0.21
NH_4_^+^	mg L^−1^	0.04	0.32	0.15	0.07	0.18	1.03	0.61	0.22
NO_3_^−^	mg L^−1^	0.01	0.34	0.12	0.07	BDL	4.09	0.85	1.43
Stable isotopes in water									
δD	^0^/_00_	− 85.41	− 72.55	− 79.88	5.54	− 59.06	− 41.89	− 48.99	7.16
δ^18^O	^0^/_00_	− 11.20	− 9.80	− 10.67	0.56	− 11.02	− 5.74	− 7.64	1.1
d-excess	^0^/_00_	0.83	8.25	5.47	2.78	2.89	29.10	12.15	10.75
Metal concentrations in water									
Cu	mg L^−1^	BDL	0.386	0.036	0.089	0.178	0.556	0.293	0.068
Zn	mg L^−1^	BDL	0.534	0.109	0.102	0.015	1.065	0.151	0.189
Fe	mg L^−1^	1.375	3.950	2.294	0.556	0.240	67.480	6.507	11.348
Co	mg L^−1^	BDL	0.046	0.003	0.009	0.179	0.351	0.261	0.063
Mn	mg L^−1^	0.096	0.306	0.164	0.052	BDL	0.238	0.042	0.047
Cd	mg L^−1^	0.016	0.077	0.026	0.011	BDL	0.025	0.003	0.005
Pb	mg L^−1^	BDL	BDL	BDL	BDL	BDL	0.097	0.003	0.016
Cr	mg L^−1^	0.007	0.541	0.324	0.136	BDL	0.424	0.118	0.135
Se	mg L^−1^	3.445	8.559	6.236	1.153	2.784	6.47	5.522	0.640
Ba	mg L^−1^	BDL	0.996	0.074	0.199	BDL	0.536	0.031	0.102
Metal concentrations in suspended solids									
Co	mg kg^−1^	651	1650	1145.4	426.5	1287	2389	1879.6	416.5
Cu	mg kg^−1^	752.3	1545	1026.6	322.3	187.6	854.2	548.6	315
Mn	mg kg^−1^	785.5	1573	1158.8	386.7	1730	2902.5	2321.8	503.5
Zn	mg kg^−1^	158.5	279.5	232.6	60	111.5	188.5	141.3	30.7
Se	mg kg^−1^	86	436	225.7	142.4	14	293	212.4	112.8
Fe	mg kg^−1^	33,430	46,890	38,244	5291.8	23,340	37,610	29,116	6054.7
Al	mg kg^−1^	17,310	21,045	19,282	1609.6	4335.5	10,670	7645.1	2533.2
Cd	mg kg^−1^	4.7	12.75	6.68	3.45	3.55	6.15	4.34	1.1
Ba	mg kg^−1^	507.5	279.5	826.4	502.6	563	1601.5	1059.8	452.1
Metal concentrations in sediments									
Co	mg kg^−1^	796.5	2337.2	1318.11	355.27	1331.4	2902.9	1969.36	419.82
Cu	mg kg^−1^	76.8	3901.3	1050.78	764.46	134	952.9	355.67	224.93
Mn	mg kg^−1^	38.7	523.3	174.19	118.31	39	399.30	163.23	89.51
Pb	mg kg^−1^	BDL	11.4	3.35	3.36	BDL	0.2	0.01	0.03
Zn	mg kg^−1^	86.4	213.5	133.97	31.91	91.6	197	127.59	30.48
Se	mg kg^−1^	527.7	1081.1	789.87	138.36	494.3	931.6	744.01	112.78
Fe	mg kg^−1^	33,205.7	90,397.5	52,496.3	15,269.24	27,673.6	65,400.8	39,527.7	10,870.18
Al	mg kg^−1^	16,651.5	227,258.1	110,824.88	54,123.51	1862.4	102,302	27,635.87	33,237.45
Cd	mg kg^−1^	0.99	1.74	1.25	0.19	0.96	1.53	1.18	0.14
Cr	mg kg^−1^	29.7	113.8	92.63	22.99	99.3	113.3	110.96	2.15
Ba	mg kg^−1^	774.1	7511.3	2301.48	1569.24	210.3	3186	1487.73	848.29

*BDL*, below detection limit

### Controlling mechanisms

**Fig. 3:**
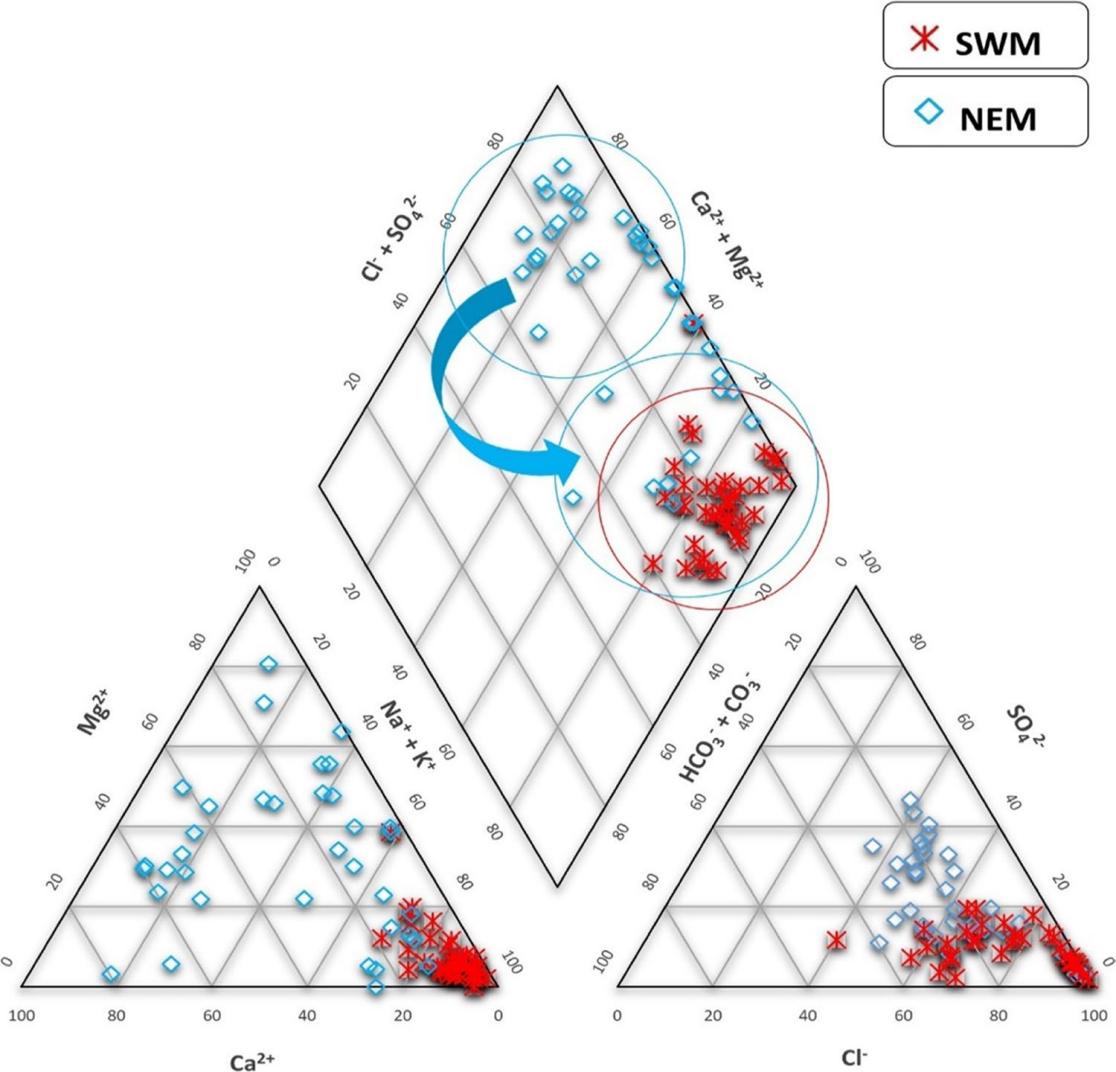
Piper plot representation of water during SWM and NEM

During SWM, all the samples fall under the Na-Cl type of water in the Piper plot and are directly influenced by seawater in the estuarine region (Fig. 3). This phenomenon can be explained by the variety of conditions like gentle coastal hydraulic gradients, tidal and estuarine activity, sea level rises, low infiltration, excessive withdrawal, and local hydrogeological conditions (Sivasubramanian et al. 2013; Senthilkumar et al. 2017). In this study, the Na-Cl type of water is dominating because of the high tidal influence, considering the time of sample collection along with the low flow of the Sibuti River system due to less rainfall during the SWM period. However, in the diagrams for NEM, samples fall under various facies including Na-Cl type, Ca–Cl type, and Ca–Mg–Cl type, unlike the SWM. The dominant water type in NEM can be represented as Ca–Cl type > Na-Cl type > Ca–Mg–Cl type (Fig. 3). Samples falling in the Ca–Cl type are prominent at the most upstream side of the estuary, which indicates the weathering of limestones and dolomites in the catchment areas or direct recharge from rainwater. During the phase of seawater intrusion, underneath the freshwater flow, there is an initial increase in salinity and a rapid and marked reverse exchange of Na/Ca, which is recognized by the characteristic Ca–Mg–Cl facies (Ravikumar and Somashekar 2017). This type of water evolves towards facies that are closer to seawater (Na-Cl). This coastal region possibly represents ion-exchange reactions or a hydrochemical evolutionary path from Ca–Cl and Ca–Mg–Cl water type to Na–Cl water type. Similar observations were confirmed by indices of base exchange, where 30 samples showed an indication of base ion exchange in water, whereas 6 samples in the same condition showed reverse ion exchange. On the contrary, during NEM, this process is opposite and showed 33 samples under exchange between Na^+^ and K^+^ in water with Mg^2+^ or Ca^2+^ in rock and is an indication of reverse ion exchange. Considering the sharp increase in the concentration of Ca^2+^ and Mg^2+^ during NEM, it is an indication of the mixing of various weathered ions in river water through runoff from its catchment, which mainly consists of sandstones and calcareous sandstone, shale, limestone, and marl and are dominant in Sibuti and Lambir formations (Nagarajan et al. 2015, 2017a). These rocks are prominent to weathering and consist of calcite (CaCO_3_), dolomite (CaMg (CO_3_)), and less prominent feldspars along with weathering resistant minerals like quartz (Nagarajan et al. 2015; Simon et al. 2014). On the other hand, the increase in the concentration of Cl^−^ from SWM to NEM is also attributed towards the base exchange of Na^+^ for Ca^2+^ and Mg^2+^ as mentioned earlier.

**Fig. 4:**
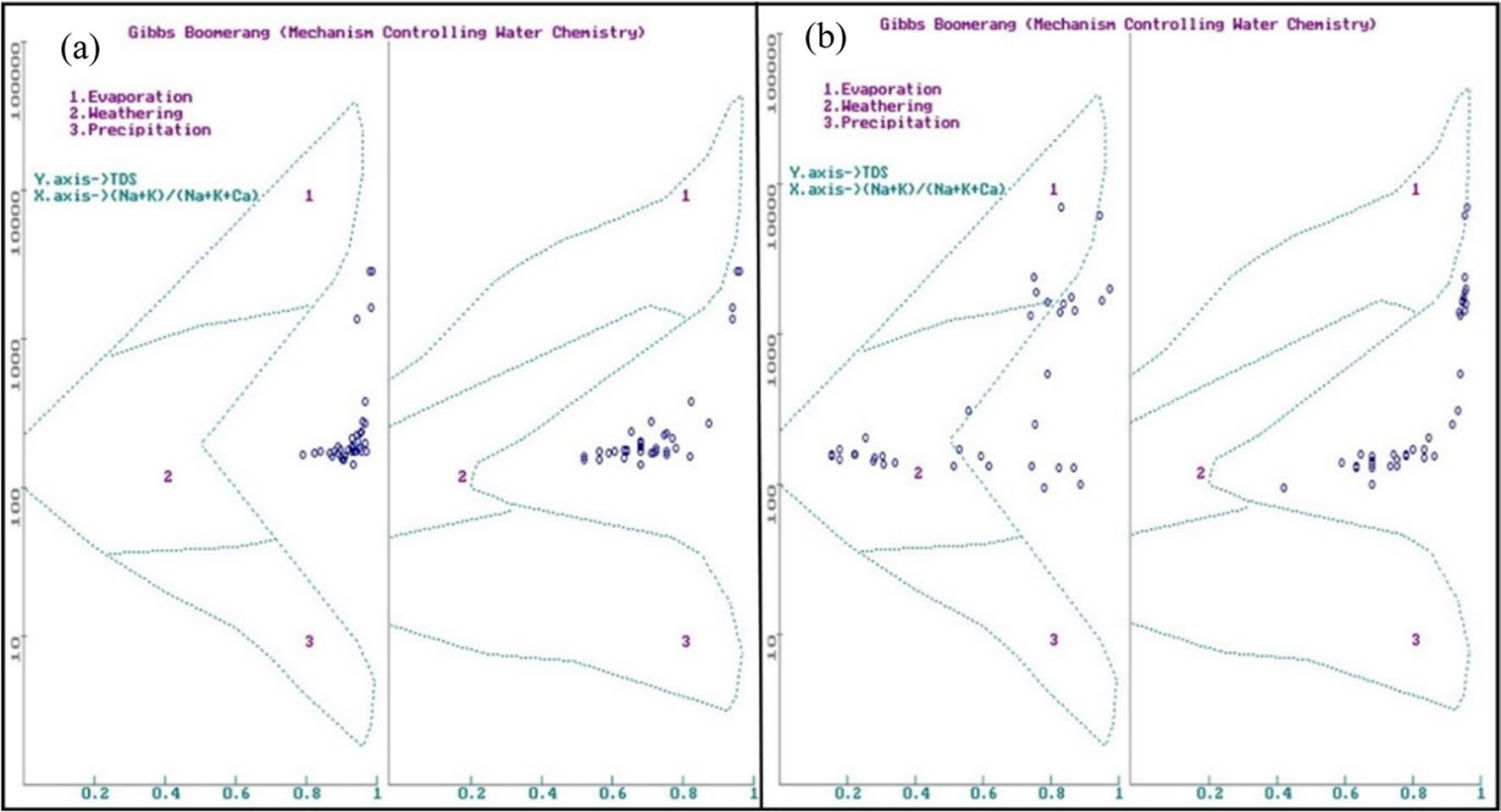
Gibbs classification of water samples according to mechanisms controlling the water chemistry during SWM (a) and NEM (b)

Samples in Gibbs plot indicated an integrated mechanism of high weathering, low evaporation, and precipitation along with input from other sources (Annapoorna and Janardhana 2015). These sources might include the effect of seawater intrusion because of the tides along with the agricultural run-off, during SWM . In contrast, NEM has shown a major variation in the plot by the representation of samples in weathering zone due to rock-water interaction and also in evaporation zone. The majority of the samples in the cation plot falls in the rock weathering field, which may be due to the weathering in the upstream and downstream regions of the river as discussed earlier. On the contrary, the rest of the cations and all the anions fall outside the defined zone (Fig. 4), indicating an additional anthropogenic similar mechanism during SWM.

### Saturation index (SI)

**Fig. 5:**
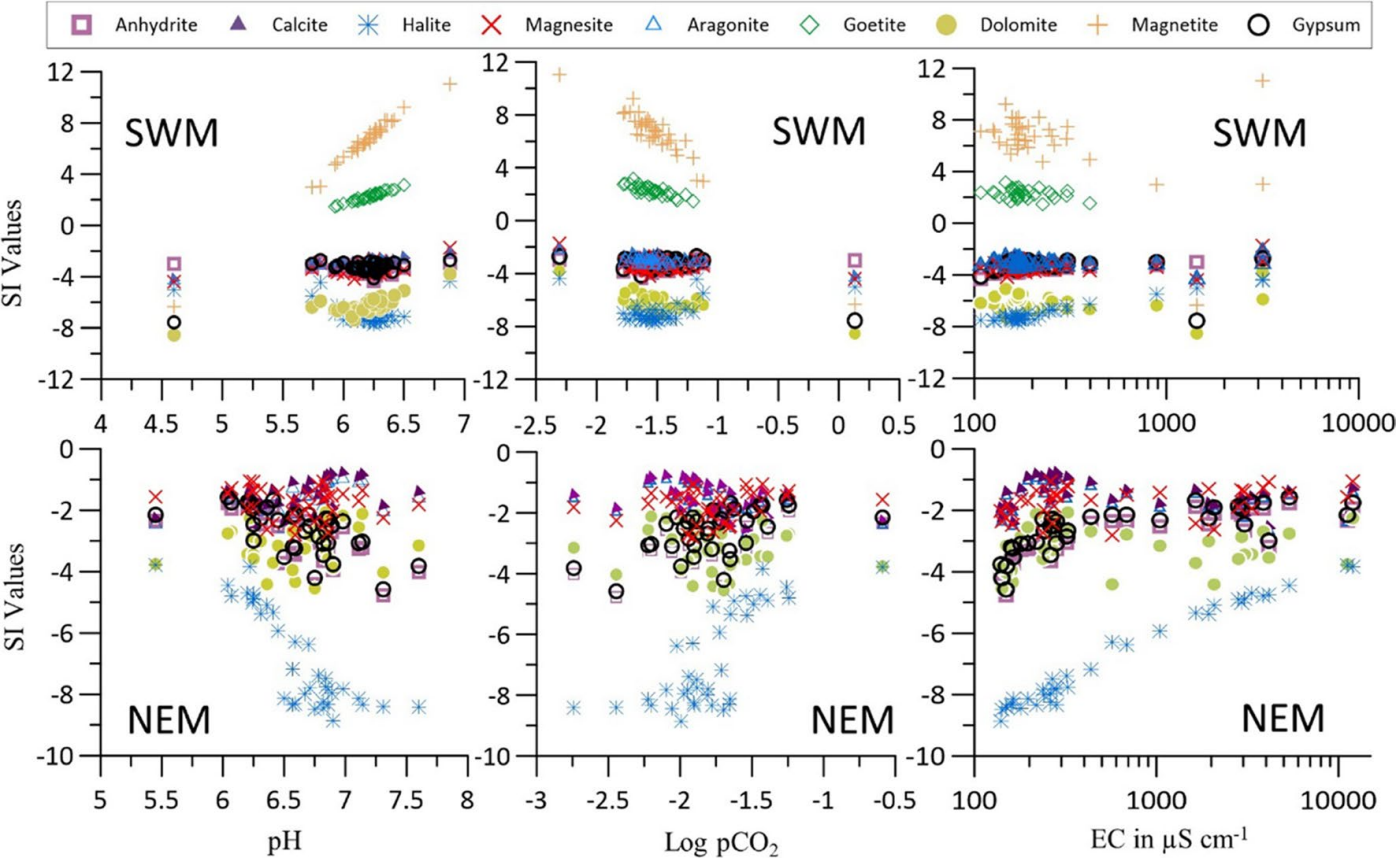
Relationship of log pCO_2_, pH, and EC with SI of minerals

In both seasons, carbonate, sulfate, and halide groups of minerals were found to be in undersaturated condition in the estuary except for oxide and oxyhydroxide group of minerals such as magnetite and goethite, which were found to be in over saturated condition during SWM (Fig. 5). The log pCO_2_ was found higher than the atmospheric equilibrium (− 3.5) (Prasanna et al. 2010; Srinivasamoorthy et al. 2014). In such a case, respiration of organic matter and dissolution of carbonate minerals play a major role in the increase of CO_2_. Meanwhile, pH in the estuary is inversely related to log pCO_2_. These values indicate a higher residence time of river water during SWM due to the low flow of the river, whereas lower log pCO_2_ values during NEM are an indication of the freshwater recharge because of higher rainfall during this monsoon. The absence of any definite trend of EC with carbonate (calcite, magnesite, aragonite, and dolomite) and sulfate minerals (gypsum and anhydrite) (Fig. 5) suggests the negligible influence of seawater in the dissolution of these minerals. The higher dissolution of carbonates is due to the higher residence time of water coupled with degassing of CO_2_ (Chidambaram et al. 2011) in water. This was confirmed by an observed lower average value of log pCO_2_ during SWM. The dissolution of sulfate minerals was found to be more aligned and falling along the recharge line of the river during NEM (Fig. 5). Higher fresh recharge, higher discharge, abundance of TSS, higher pH, and lower log pCO_2_ in the upper part are also responsible for higher dissolution of sulfate minerals during NEM. The dissolution is highest for halite than any other minerals considered in the current study, which may be due to its nature of high solubility (Klimchouk et al. 1996; Naderi et al. 2016). It responds to EC during both seasons perfectly with dissolution decreases with higher EC values. During SWM, higher dissolution takes place between 100 and 200 µS/cm of EC, whereas dissolution is well spread during NEM due to reducing saline water influence through freshwater recharge. On the other hand, SI values of halite relate significantly with log pCO_2_ and pH during NEM, indicating a lower residence time of saline water (Prasanna et al. 2010; Naderi et al. 2016) while an increase in dissolution in the seawater flow direction (towards upstream direction). This is due to the intensive mixing and ion exchange process caused by the dissolution of gypsum and anhydrite (Hamzaoui-Azaza et al. 2013; Juen et al. 2015; Naderi et al. 2016) and validates the Ca–Mg–Cl type of water observed during NEM, whereas the domination of Na-Cl type of water ruled out such ion exchange processes (Juen et al. 2015). Fe oxides are found to be precipitating during SWM, whereas such precipitation/dissolution is uncommon in NEM water samples. These oxides show an increasing trend with an increase in pH and a decrease in log pCO_2_ values of water in the estuary (Fig. 5). The supersaturation state of magnetite is responsible for lowering the concentration of dissolved Fe in estuarine water. Both oxide and oxyhydroxides approach saturation with a decrease in pH condition, attributed to the changes in redox conditions, pH, and hydrolysis reactions (Mapoma et al. 2017). According to Tosca et al. (2019), effluent-seawater mixing with river water strongly reduces the flux of dissolved Fe into the sea due to the formation of oxides and oxyhydroxides. These observations suggest that the precipitation of Fe is mainly associated with the residence time of river water for a longer period during SWM as compared to NEM (Fig. 5). This might be due to increased tidal resistance observed in the river and low flow (seasonal fluctuation) which is allowing Fe to precipitate (Mapoma et al. 2017) in the estuary in the form of magnetite and goethite.

### Statistical evaluation

Factor analysis was carried out for all the parameters in water, sediments, and suspended solids for both seasons to identify the underlying geochemical processes and sources. The varimax rotation utilized for factor analysis for both seasons and the rotated component matrix is presented in Tables 2 and 3 for water, Table 4 for SS, and Tables 5 and 6 for sediments. The components having eigen values > 1 were considered for interpretation. The factor analysis for water explained 82.27 and 82.95% of variance during SWM and NEM (Tables 2 and 3), respectively, and the factor analysis for suspended solids explained 95.17 and 93.62% of variance (Table 3) for both seasons. Similarly, factor analysis carried out for sediments explained 72.65 and 72.87% of the variance (Tables 5 and 6).

**Table 2:**
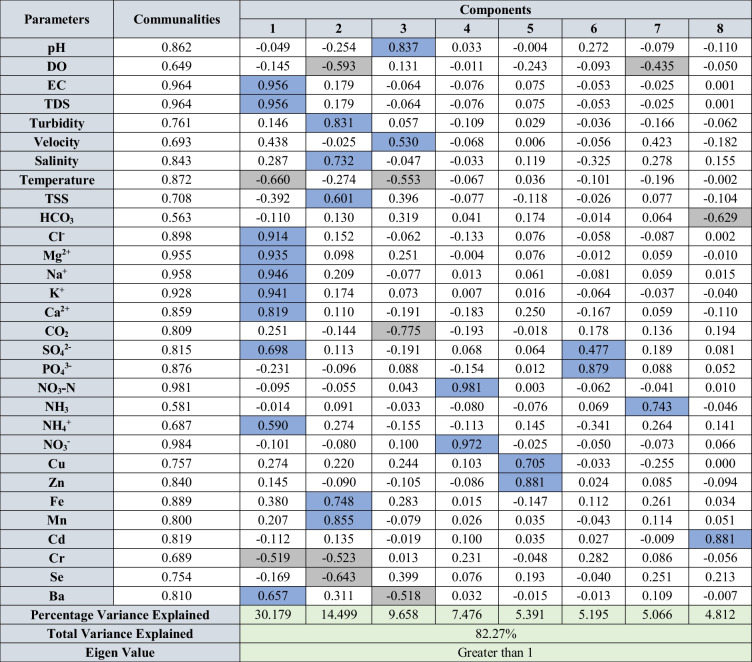
Rotated component matrix of water contained physico-chemical parameters, major ions, nutrients, and trace metals during SWM

Blue cells: positive factor loading; grey cells: negative factor loading

**Table 3:**
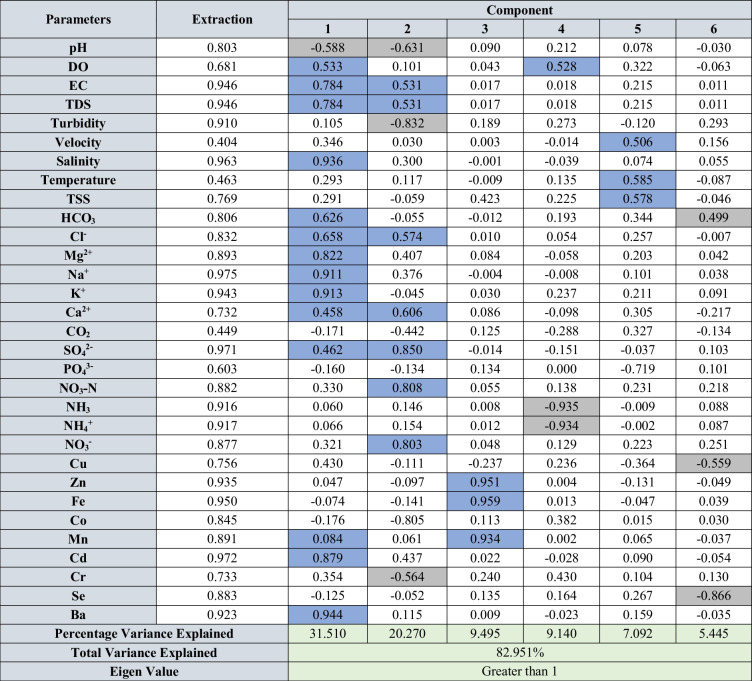
Rotated component matrix of water contained physico-chemical parameters, major ions, nutrients, and trace metals during NEM

Blue cells: positive factor loading; grey cells: negative factor loading

**Table 4:**
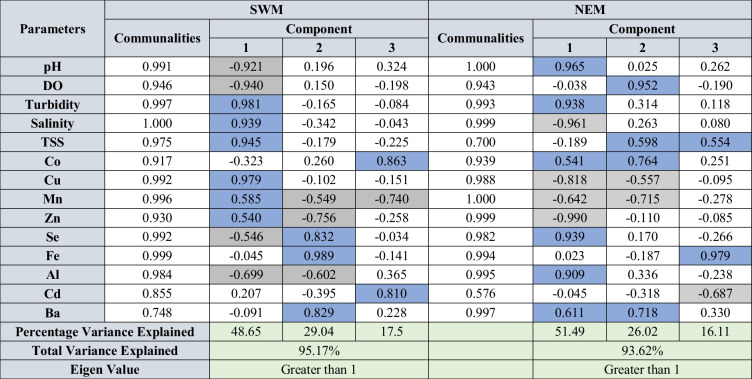
Rotated component matrix of trace metals in SS during SWM and NEM

Blue cells: positive factor loading; grey cells: negative factor loading

**Table 5:**
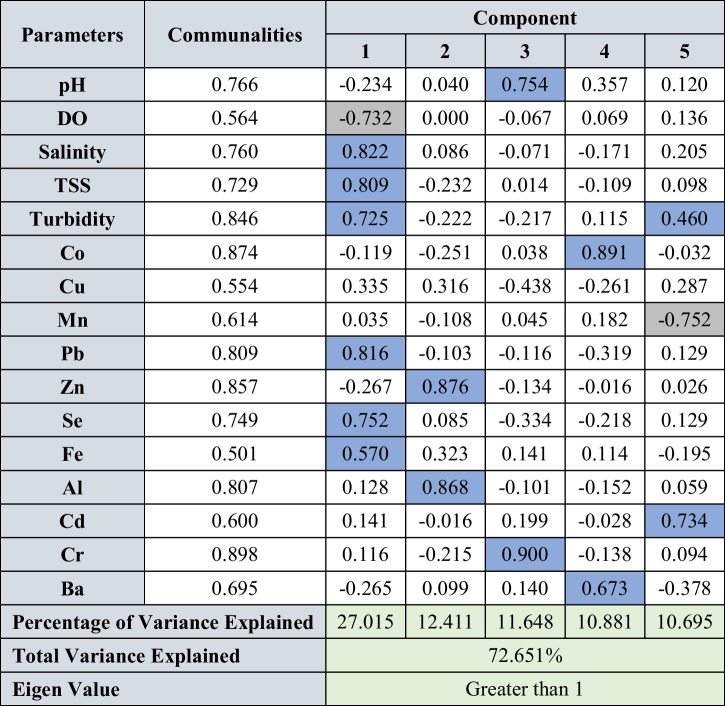
Rotated component matrix of metals in sediments during SWM

Blue cells: positive factor loading; grey cells: negative factor loading

**Table 6:**
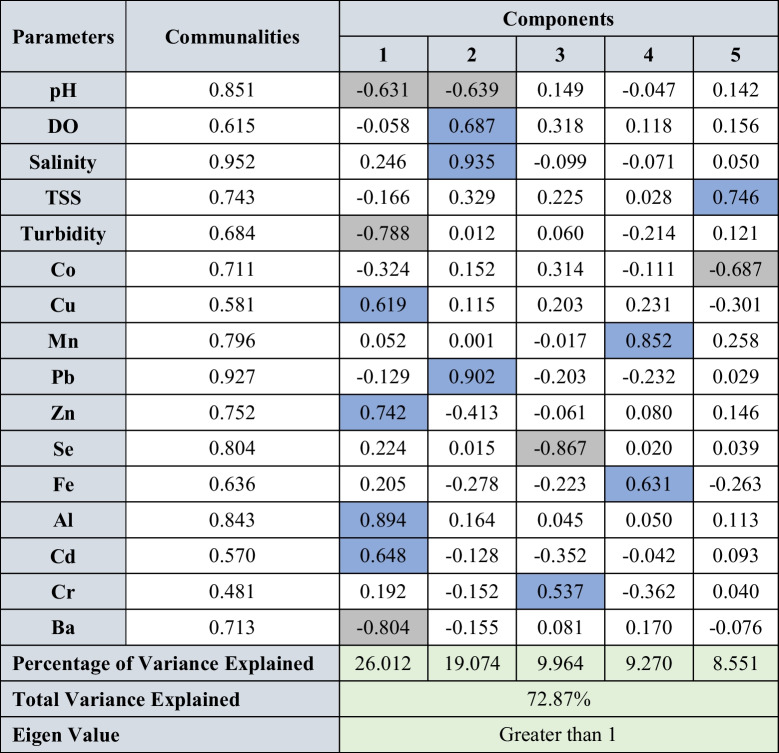
Rotated component matrix of metals in sediments during NEM

Blue cells: positive factor loading; grey cells: negative factor loading

#### Geochemical mechanisms in water

##### Southwest monsoon (SWM)

**Fig. 6:**
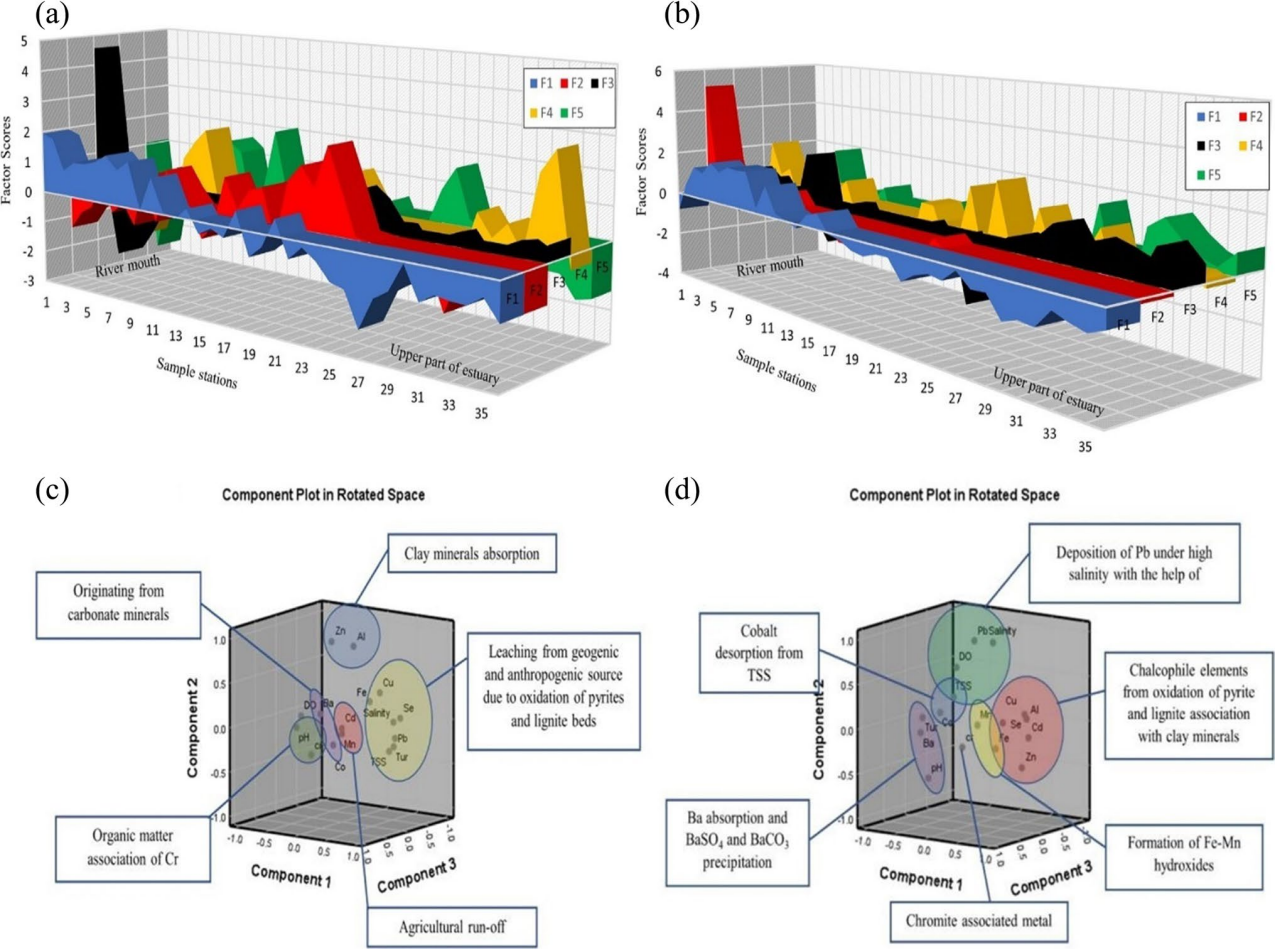
Graphical representation of factors scores (**a** SWM; **b** NEM) and dominating principal components (**c** SWM; **d** NEM) for sediments

**Fig. 7:**
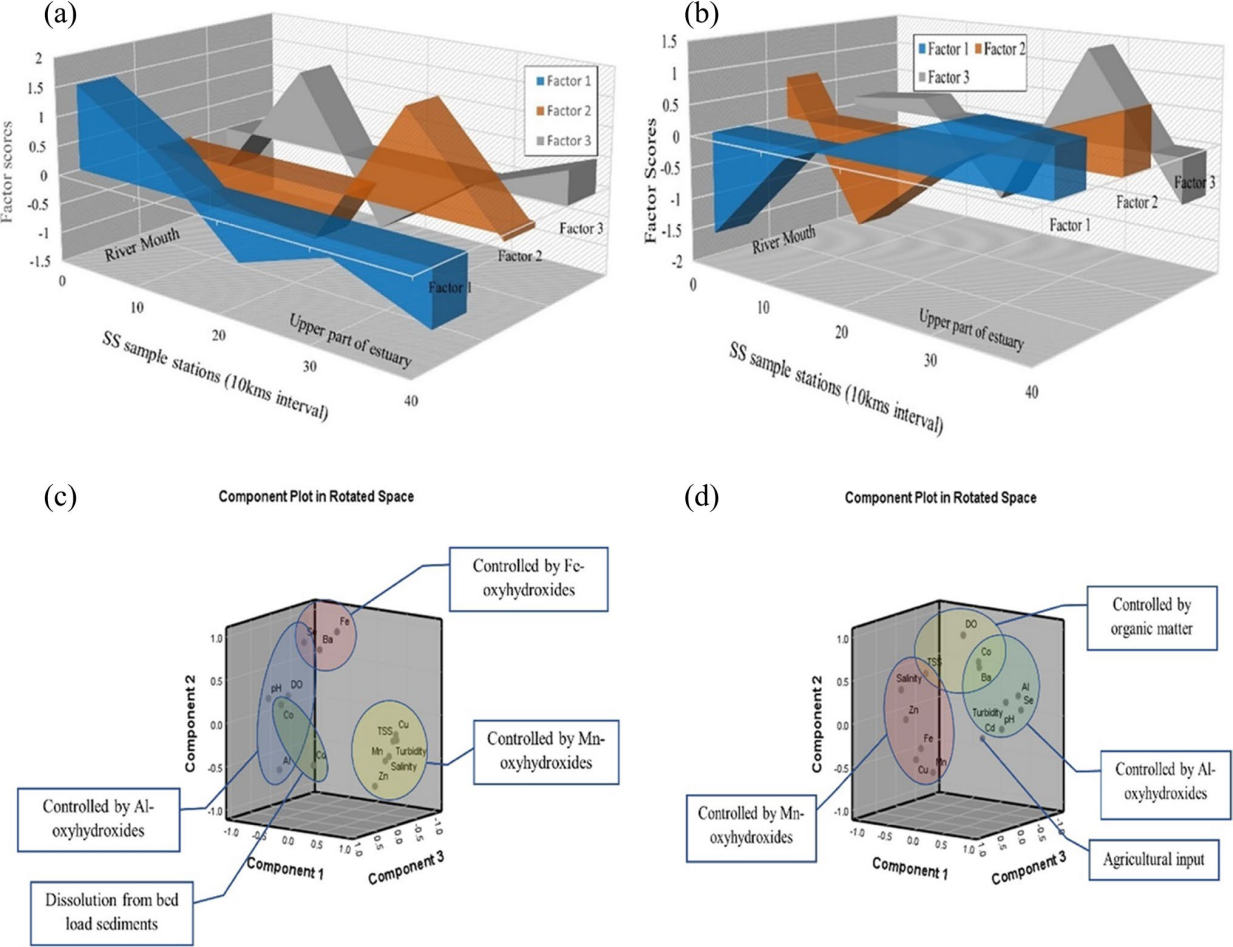
Graphical representation of factors scores (**a** SWM; **b** NEM) and dominating principal components (**c** SWM; **d** NEM) for suspended solids

**Fig. 8:**
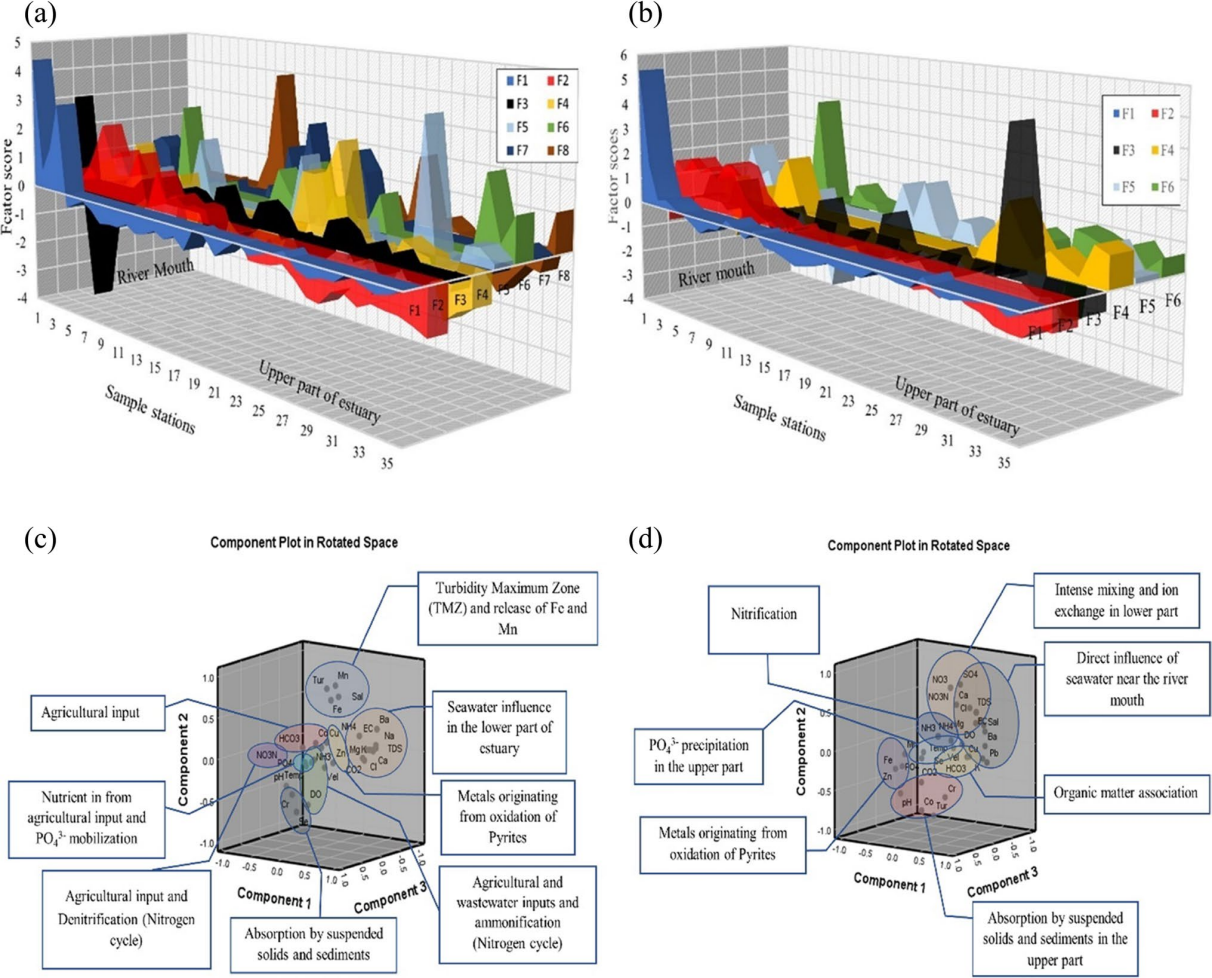
Graphical representation of factors scores (**a** SWM; **b** NEM) and dominating principal components (**c** SWM; **d** NEM) for water

The 8 factors during SWM with eigenvalues more than one were considered. Factor 1 has major EC, TDS, velocity, Cl^−^, Mg^2+^, Ca^2+^, Na^+^, K^+^, SO_4_^2−^, NH_4_^+^, and Ba. This factor has a major association at station 1 with a factor score of 4.383 (Fig. 6a). The association and trend of these parameters in water indicate the domination of seawater in the estuary during SWM (Patra et al. 2012). Factor 2 is loaded with turbidity, salinity, TSS, Fe, and Mn, where a negative loading of DO, Se, and Cr is also associated with the process (Fig. 7c). This indicates the tidal-induced turbidity in the estuary initiating resuspension of suspended solids (Uncles et al. 1985) and reveals the injection of Fe and Mn into the water column from the hydroxide phases available in the sediments and suspended solids (Turner and Millward 2000; Callaway et al. 1988). This factor is dominant in the mid zones of the estuary (stations 6 to 13; Fig. 8a) and the association of TSS indicates particulate organic matter association of sediments and suspended solids with the formation of cation-induced coagulation of negatively charged humic colloids containing Fe and Mn in this zone. This forms organic Fe and Mn complexes in a water solution with a reduction of DO in water (Shapiro 1964; Sholkovitz 1976; Boyle et al. 1977; Mayer^1982^; Zhou et al. 2003; Jilbert et al. 2016; Wen et al. 2019) and might be responsible for absorption of Se and Cr from the water column (Bewers and Yeats 1978; Campbell and Yeats 1984). The positive loading of pH with negative loading of CO_2_ and Ba in factor 3 indicates the reaction of CO_2_ with seawater to cause respiration of organic matter with the generation of various organic acids including the formation of carbonic acid in water, making the water acidic (Eq. 6) (Mook and Koene 1975; Mucci et al. 2011; Saifullah et al. 2014; Van Dam and Wang 2019).

6 $${\mathrm{H}}_{2}\mathrm{O }+\mathrm{ C}{\mathrm{O}}_{2}\iff {\mathrm{H}}_{2}{\mathrm{CO}}_{3}(\mathrm{carbonic\ acid})\iff {\mathrm{H}}^{+}+{\mathrm{HCO}}_{3}^{-}$$

NO_3_^−^ and NO_3_-N loading in factor 4 and high factor scores near stations 6, 23, 24, 25, and 26 (Fig. 6a), which are near various agricultural channels and tributaries such as Sungai Kejapil indicate towards the leaching of these nutrients from the adjacent agricultural and anaerobic denitrification processes in the estuary (Haaijer et al. 2006). On the other hand, factor 7 represents NH_3_ with a weak negative loading DO which is an indication of the combined effect of ammonification and NO_3_ reduction to NH_4_^+^, where DON (dissolved organic nitrogen) and NO_3_ give rise to NH_4_^+^ (Scott et al. 1999). This process is continuing to form NH_3_ with the consumption of available DO in the water column (Müller et al. 2018). The major loading of chalcophile metals such as Cu and Zn in factor 5 is mainly associated with the oxidation of pyrites (Anandkumar et al. 2022), which are widely available in Sibuti and Lambir formations (Nagarajan et al. 2015, 2017a). These metals are not associated with any physico-chemical parameters indicating the release of such metal from the source rocks due to chemical weathering. Factor 6 is loaded with PO_4_^3−^ and weak loading of SO_4_^2−^ and has high factor scores at stations nearer to agricultural fields (station nos.: 7, 17, 20, 26, 34, and 36) (Fig. 7a) indicating PO_4_^3−^ input from agricultural runoff water (whereas prevailing freshwater Hartzell and Jordan 2012; Nystrand et al. 2016). Station 26 reported a higher loadings for both factors 4 and 6 along with a decrease in DO concentration at this station (highest DO was reported at the station 27) (Fig. 7a). This observation and weak loading of SO_4_^2−^ in this factor indicate the process of denitrification coupled to sulfide oxidation and organic matter respiration with consumption of DO at station 26. This process leads to an increase in PO_4_^3−^ mobilization (e.g., Lamers et al. 2002; Boomer and Bedford 2008). Factor 8 has major loading of Cd along with negative loading of HCO_3_^−^. Despite being a chalcophile group of elements, independent behaviour of the metal in this component indicates the leaching of Cd from agricultural fields as phosphate-based fertilizers are widely used in agricultural fields and are the major source of Cd in Malaysian rivers (De Boo 1990). This practice is common in Borneo where these fertilizers provide maximum growth to palm oil plantations in peat-based soil (Zaharah et al. 2014). The factor score for this component is higher at stations 13, 14, 26, and 36 (Fig. 7a). Considering the land use map (Fig. 2), the proximity of these stations (13, 14, and 36) is very close to the agricultural fields situated near the estuary, which further validates the source of such metal in estuarine water.

##### Northeast monsoon (NEM)

The factor analytical results of water from NEM are summarized in Table 3 and consist of 6 major components. Factor 1 is during NEM has major loading of EC, TDS, DO, salinity, HCO_3_^−^, Cl^−^, Mg^2+^, Ca^2+^, K^+^, SO_4_^2−^, Cd, Ba, and Cr along with negative loading of pH (Fig. 7b). The association of the mentioned parameters indicates saline water-influenced processes like SWM despite the large infusion of freshwater during NEM from the riverine side. The factor scores of 5.36 and 1.62 at stations 1 and 2 (Fig. 7b) reveal the direct influence of seawater in the lower part of the estuary. The loading of HCO_3_^−^ with Cl^−^, Mg^2+^, and Ca^2+^ is illustrative of their contribution from seawater as HCO_3_^−^ presence in natural water varies from pH 4.5 to 8.3, and the dissolution of these ions is ensured by the acidic condition near the mouth and indicated by negative pH loading. Positive loading of Cd and Ba attributes to the immediate increase in salinity near the mouth and is giving rise to the concentration of Ba (Coffey et al. 1997) and Cd (Greger et al. 1995) from the suspended particles and sediments. Factor 2 has major loading of EC, TDS, Mg^2+^, Cl^−^, Ca^2+^, SO_4_^2−^, and NO_3_^−^ and has a negative affinity towards pH, Co, Cr, and turbidity, which contributes 20.27% of the total variance. This factor indicates the intense mixing of fresh water in the lower region of the estuary, where high factor scores observed from stations 2 to 9 (Fig. 7b) have a higher affinity with this component. The association of EC, TDS, Mg^2+^, Cl^−^, and Ca^2+^ indicates towards ion exchange process (Thivya et al. 2015) in the estuary (Fig. 7d). Regarding negative loading of Co and Cr and turbidity, these metals are removed from the water column and finding their way to the sediments with an increase in the influence of Cl^−^ (seawater). Under such conditions, Cr forms a hexaaquo complex ([Cr (H_2_O)_6_] Cl_3_). These hexaaquo complex and Cr-organic associations are stable in freshwater but destabilize with an increase in ionic strength and precipitate as floccules (Pađan et al. 2019; Campbell and Yeats 1984). Due to the intensive mixing, DO carried by river water plays a major role in the positive loading of both SO_4_^2−^ and NO_3_^−^. Sulfides under reoxygenated conditions are oxidizing to sulfates and remineralization of organic matter gives rise to more SO_4_^2−^ in the water column (Patra et al. 2012; Matson and Brinson 1985; Malcolm et al. 1986). In addition, the nitrification process (nitrogen cycle) increases with the availability of DO to produce more NO_3_ in the water column from NH_3_ and NH_4_^+^. Such a process gives rise to more H^+^ ions and acidic conditions in water, which justifies the negative loading of pH in factor 2 (Müller et al. 2018; Scott et al. 1999). The negative loading of NH_3_ and NH_4_^+^ in factor 4 validates this reaction (Eq. 7), and high positive loading of DO is observed at the same stations while representing 9.14% in variance.

7 $${\mathrm{NH}}_{3}+{\mathrm{H}}_{2}\mathrm{O}+2{\mathrm{O}}_{2}\iff {\mathrm{NH}}_{4}^{+}+{\mathrm{OH}}^{-}+2{\mathrm{O}}_{2}\iff {\mathrm{NO}}_{3}^{\_}+2{\mathrm{H}}_{2}\mathrm{O}+{\mathrm{H}}^{+}$$

As NH_3_ and NH_4_^+^ are the by-products of the degradation of organic matter under anaerobic conditions (Canfield et al. 1993; Baric et al. 2002), an increase in DO eliminates the enrichment of NH_3_ and NH_4_^+^ in the estuary. The factor scores at stations 9 (2.001) and 29 (2.162) (Fig. 7b) suggest that nitrification (nitrogen cycle) is a dominating process during NEM mainly in the upper part and mixing zone of the estuary. Factor 3 has positive loading of Fe, Mn, and Zn, and the independency of this parameters with pH attributes towards the source of these metal association to be terrestrial in origin and the run-off might be the carrier of these metals during NEM as terrestrial input. As the metal association is mainly composed of chalcophile elements, oxidation of pyrites might be the major source of these metals in estuarine waters (Galán et al. 2003; Lu et al. 2005; Nieto et al. 2007; Fernandez and Borrok 2009; Chopard et al. 2017). The higher factor scores in the upper part of the estuary (station 32) (Fig. 7b) imply that the source is mainly from the Sibuti Formation, where pyrite concretions are common (Nagarajan et al. 2015, 2017a). On the other hand, higher run-off during NEM does not permit the saline water to impact the association in the upper part of the estuary. Factor 5 is loaded positively with temperature, TSS, and velocity along with high negative loading of PO_4_^3−^. PO_4_^3−^ remobilization in estuary mainly happens under reducing conditions (Deborde et al. 2007). The absorption of PO_4_^3−^ by sediments happens under an increase in temperature with decreasing salinity conditions (Zhang and Huang 2011). During NEM, infusion of freshwater rich in DO decreases the reducing conditions, whereas resistance provided by such freshwater input towards saline water input can be validated by positive loading of velocity from the riverine side. Positive loading of temperature and negative loading of PO_4_^3−^ indicate the sorption of PO_4_^3−^ under such conditions where it is finding its way to suspended solids and sediments. The factor scores suggest this process to be dominant in the upper part of the estuary. On the contrary, stations 2 and 4 are observed to have higher factor scores (Fig. 7b), which is mainly because of the higher turbidity and concentration of DO observed in these stations. Factor 6 has positive loading of HCO_3_^−^ and negative loading of Cu and Se. The negative loading of Cu might be due to the formation of insoluble [Cu (HCO_3_)_2_] and association with organic compounds that exist in colloidal form favored by an increase in HCO_3_^−^ concentration in water (Drogowska et al. 1994; Namieśnik and Rabajczyk 2010) while removing it from the water column. Similarly, under well-oxygenated conditions, sorption of the dissolved form of Se such as SeO_3_^2−^ and SeO_4_^2−^ on sediments and suspended solid happens in aqueous conditions (Kieliszek 2019; Hung and Shy 1995), and such retention is closely linked with the presence of organic matter in water (Söderlund et al. 2016). This process is dominant in the lower part of the estuary (Fig. 7b).

#### Geochemical mechanisms of suspended solid (SS)

##### Southwest monsoon (SWM)

Factor 1 has strong positive loading (> 0.5) of Cu, Mn, and Zn along with saline water-induced parameters like salinity, turbidity, and SS along with negative loading of pH, DO, Al, and Se. This component shows the highest positive factor score near the river mouth and the lowest in the upper part (Fig. 7a). The negative loading of Al and Se along with pH and DO indicate the absorption of Se by Al-oxyhydroxides under high pH and high DO conditions due to their reactive surfaces (Hsu 1989; Jiann and Ho 2014; Hao et al. 2020). The low negative factor scores at stations 20 and 40 km confirm such absorption in the upper part of the estuary (Fig. 7a). On the other end, the positive loading of Cu, Mn, and Zn indicates towards the release of these metals from pore water mainly due to density stratification as a result of high salinity gradient, which causes organic respiration and maintains an acidic environment near the sediment–water interface (Turner and Millward 2000). This condition is confirmed by the negative loading of both pH and DO in this factor. These released metals are being absorbed with the help of Mn-oxyhydroxides formation on the ambient and diluent SS while replacing Fe and Al-hydroxides formed in the upstream section. In the presence of organic acids generated from their respiration, Mn hydroxides have more reactive properties than Al hydroxides (Qin et al. 2018; Habibah et al. 2014), which might be the reason behind such absorption. With this effect, Al and Fe are removed from the water column by the aggregation and precipitation of diaspore in distinct flocculation zones because of increasing ionic strength, as the water is subjected to steep pH and salinity gradients (Ferguson and Eyre 1999), whereas Se goes to dissolved phase with such effect and selecting an increase in water during SWM. This process is dominant in the lower part of the estuary (Fig. 7c). Factor 2 has major loading of Se, Fe, and Ba; meanwhile, there is a significant negative loading of Al, Mn, and Zn. The positive association of Fe, Se, and Ba indicates the absorption of these metals by Fe-oxyhydroxides (Zhang et al. 2014), and a high positive factor score is observed at the 30-km station (Fig. 7a). As Fe is the dominating trace metal in the SS, the particulate Fe might indicate the presence of Fe (OH)_3_ and Fe (OH)_4_^−^ (Ferguson and Eyre 1999). The tendency of Ba (Mori et al. 2019) and Se (Hung and Shy 1995; Kieliszek 2019) to form oxides with Fe in better-oxygenated conditions far away from the sea is giving rise to the increase in the concentration of these metals in the estuary, which eventually decreases towards the river mouth. The negative loading of Al, Zn, and Mn has been in the intermediate zone of the estuary, which is confirmed by the peak negative factor score from the station at 20th to 30th km (Fig. 7a). This part is the mixing zone with the partial influence of freshwater and saltwater, and such association suggests the absorption of Zn by both ambient Mn-oxyhydroxides and diluent Al-oxyhydroxides. It works as a buffer zone for both oxyhydroxides as the river mouth is dominated by Mn-oxyhydroxides due to organic respiration and acidic condition additionally by significant influence of saltwater-induced turbidity, while the upper part is mainly dominated by Al-oxyhydroxides as discussed earlier, due to ambient DO and higher pH in factor 1. Factor 3 shows positive loading of Co and Cd along with negative loading of Mn. The high positive factor score is observed at a 10-km distance from the river mouth (Fig. 7a). This station is also associated with a major spike in the concentrations of Co and Cd during this season. As discussed before, the decisive factor behind the control of Co into the water column is bedload sediments rather than suspended particulate matter in the estuary. The majority of Co occurs mainly in a non-reactive form and is buried with accumulating sediments (Gendron et al. 1986). In the case of Cd, particulate Cd tends to settle at the sediment surface that is mostly bound to biogenic material present on it (Boyle et al. 1976; Gendron et al. 1986). But both these metals follow a redox-sensitive pattern of dissolution in the reducing zone of the sediments leading to vertical migration into the water column and enrichment by precipitation in the oxidized surface layer. In addition, these metals have an affinity towards Mn-hydroxides during redistribution in particulate form (Gendron et al. 1986). The formation of particulate Mn hydroxides in reducing zones is evident from the earlier discussion to unravel the process governing factor 1. The association of Co and Cd from bed sediments and Mn hydroxide presence as particulate matter might be responsible for the negative loading of Mn. The released dissolved Co and Cd are being absorbed by the Mn hydroxides in the water table (Fig. 7c), which explains this association and the spike in concentration observed at station 2 (10 km) (Fig. 7a).

##### Northeast monsoon (NEM)

Factor 1 has a strong loading of Co, Se, Al, Ba, turbidity, and pH along with negative loading of salinity, Cu, Mn, and Zn, which explains 51.49% of the total variance (Fig. 6d). This factor is very similar to the process observed in factor-1 during SWM. The association of Al, Se, Ba, and Co indicates absorption by Al oxyhydroxides under ideal pH (4–7) levels in the upper region of the estuary. In this pH range, Al-oxyhydroxides stay in insoluble particulate form (Ferguson and Eyre 1999) and absorb metals like Co, Se and Ba due to high surface reactivity (Hsu 1989; Jiann and Ho 2014; Qin et al. 2018; Mori et al. 2019; Hao et al. 2020). But the influential region for this process in factor-1 (NEM) is higher compared to SWM. The factor scores indicate the domination of positive factor in the upper part (20 to 40 km) due to dominance of freshwater and gradually decreasing towards the station at 10 km with a gradual increase in salinity (Fig. 6b). Starting from this point, negative factor score is observed to be dominating the lower part, with the higher loading of Mn, Zn, and Cu (Fig. 6b). This part of the estuary is controlled by reducing conditions due to stratification of salt and freshwater, and the pH drops due to respiration of organic matter, which are responsible for the injection of dissolved Mn, Cu, and Zn from pore water into the water column (Turner and Millward 2000). This leads to the formation of Mn-oxyhydroxides and the absorption of metals like Cu and Zn, which was also observed during NEM (factor 1). Factor 2 has significant loading of Co, Ba, DO, and TSS along with negative loading of Cu and Mn, which explains 26.02% of the variance (Fig. 6d). This indicates the common origin of these metals and is mainly obtained from the oxidation of pyrite concretion in the source rocks and weathering of shale. All the metals obtained in this factor is reported to be available in the concretion. The DO in such a process works as an oxidant and the process is likely to take place in well-oxygenated conditions (Moses et al. 1987). The association of TSS with these metals in the absence of any controlling oxides or clay minerals indicates the absorption of Co and Ba by particulate organic matter, which are highly reactive in prevailing conditions. The higher positive factor score in the upper part supports this theory as the upper part of the estuary is observed to be dominated by freshwater (Fig. 6d). On the other hand, both Cu and Mn show a spike in trend in the lower part, and an especially significant negative factor score is obtained at station SS2 (10 km from the river mouth) (Fig. 6d). This station falls under the high mixing zone in this season and higher seawater influence leading to respiration of the mentioned organic matter, thus leading to the dissolution of Co and Ba, while making Mn oxyhydroxides more reactive with a generated organic acid in the process (Qin et al. 2018; Habibah et al. 2014) which leads to the absorption of Zn. Factor 3 has significantly higher loading of TSS and Fe and negative loading of Cd indicating the different origins of the metals, where most of the Fe concentration is geogenic and Cd concentration acquired from the SS is from anthropogenic sources like agricultural inputs. The association of TSS and Fe indicates the presence of particulate Fe in the water table, and both metals are associated with run-off water carrying SS from the river basin and agricultural fields (Pobi et al. 2019). This factor explains 16.11% of the total variance.

#### Geochemical mechanisms of sediment (< 63-µm fraction)

##### Southwest monsoon (SWM)

Factor 1 represents 27.02% of the total variance and is explained by the significant positive loadings of Pb, Se, Fe, salinity, TSS, and turbidity and a significant negative loading of DO. These elements are mainly associated with Fe-oxy-hydroxides, and their concentrations are influenced by the salinity. This indicates absorption and settlement of Se containing Fe-oxyhydroxides due to increasing seawater influence near the mouth (Fig. 6a) where Mn-oxyhydroxides dominate the absorption process as suspended solids. Such absorption was observed in the water column by SS in both water (factor-2-tidal influenced turbidity region) (Fig. 6a) and SS (Fig. 7a: upper part) factor models aforementioned. Of the positive loadings of Fe, Pb and Se in factor 1 indicates that these metals are leached from geogenic sources like pyrites and lignite beds present in the Sibuti Formation (Nagarajan et al. 2017a) and peat soil exposed within the river basin area as these elements are rich in Fe, Pb, Cu (Sia and Abdullah 2012), and Se (Yudovich and Ketris 2006; Chang et al. 2020) (Fig. 6c). Factor 2 has significant loading of Al and Zn with a variance of 12.41% in the intermediate zones during SWM (Fig. 6c). This suggests absorption of Zn on clay minerals and Al-oxyhydroxides in the intermediate zone of the estuary. Such condition was noticed in the intermediate zone of the estuary in the SS factor model (factor 2: Sect. 4.3.2.1) (Fig. 7c), where ambient Mn and freshwater carrying Al-oxyhydroxides (factor 2: Sect. 4.3.2.1) controlled the absorption in this zone and settlement of Al-oxyhydroxide containing metals with increasing seawater influence towards the mouth. This association indicates that these elements are mainly associated with clay minerals like illite, chlorite, and kaolinite in the estuarine sediments. Factor 3 has a high loading of pH and Cr with the explained variance of 11.64% during this season. In a pH range of 5–7, Cr (III) prevails and easily associates with reducible organic matter (Namieśnik and Rabajczyk 2010). The dominant species of Cr (III) in the pH range of 4.5 to 7.5 are Cr (OH)^2+^ and Cr (OH)_2_^+^ which are susceptible to bioaccumulation through suspended solids and eventually depositing in sediments (Namieśnik and Rabajczyk 2010). Such absorption was noticed in water as aforementioned in the water factor model (factor 2: Sect. 4.3.1.1), where Fe and Mn played a major role in the absorption. This independent behaviour of Cr also stipulates towards leaching of Cr from mixed sources like pyrite and shale concretion in Sibuti Formation and siliciclastic sediments of Sibuti and Lambir formations (Nagarajan et al. 2017a) rather than chromites present in NW Borneo and Sibuti Formation in the river basin. Chromites are predominantly acid-resistant in nature and do not leach Cr under the prevailing pH condition of the estuary (Weng et al. 1994, 2001).

Factor 4 has significant loading of Co and Ba, and dissociation of these 2 elements from any other elements indicates its mixed origin like non-aluminous silicate minerals where both metals are not associated with Al oxides in the sediments of Sibuti Formation (Nagarajan et al. 2019, 2017a). In addition, carbonate (calcite, dolomite, and magnesite) or Fe oxide minerals such as goethite and pyrite might be a source as well (Dehaine et al. 2021). These minerals are abundant in shale and pyrite concretion of Sibuti and Setap formations. Factor 5 has a higher loading of Cd with high negative loading of Mn. This might be due to the variation in the origin of both metals, where Cd is a major input from agricultural fields and Mn presence is mainly from geogenic sources in the study area. Moderate loading of turbidity in this component indicates the desorption of Mn hydroxide bound Cd in high turbid conditions.

##### Northeast monsoon (NEM)

Factor 1 has higher loading of Cu, Zn, Al, and Cd and high negative loading of Ba, turbidity, and pH. The chalcophile elements (Cu, Cd, and Zn) are derived from pyrite and shale concretion, and distribution is mainly controlled by clay minerals and phyllosilicates such as illite and kaolinite (Nagarajan et al. 2017a). The higher loading of Cd might be due to the absorption of Cd onto the clay minerals in the lower part of the estuary (Hao et al. 2020, Jiann and Ho 2014; Namiesnik and Rabajczyk 2010) (Fig. 6b). On the other hand, negative loading of Ba, turbidity, and pH indicate absorption. The association of Ba with clay minerals is well observed in arkoses in Sibuti and Lambir formations (Nagarajan et al. 2015, 2017a) which supports the association of Ba with clay minerals or Al-oxyhydroxides controlled factor in SS (factor 1: Sect. 4.3.2.2) and indicates settlement of such in the lower part of the estuary (Fig. 6d). The presence of Ba in Sibuti and Tukau Formations validates the leaching of this metal with chalcophile metals like Cu, Zn, and Cd. In addition, leached Ba in aquatic environments commonly precipitates as BaSO_4_ or BaCO_3_, where BaSO_4_ is mainly associated with seawater introduction because of higher SO_4_ content (Gad 2014) and respiration of organic matter, which is associated with acidic water (Marchitto 2013). The observed organic matter respiration in the water column (factor 6: Sect. 4.3.1.2) also validates the observation. Hence, formation of both precipitates are supported by absorption process in suspended solids and sedimentation with the help of Al-oxyhydroxides (Gad 2014).

Moreover, comparing the conditions during NEM, low SO_4_^2−^ concentration is observed in the upper part where the upper part has high SO_4_^2−^ content in the estuary and the turbidity of water has peaked in both ends while decreasing in the intermediate zone. In the case of pH, higher pH is observed in the upper part whereas relatively acidic conditions prevailed in the lower part. To sum up the factor with the above discussion, BaCO_3_ precipitation is evident in the upperpart and BaSO_4_ precipitation in the lower part under high turbidity conditions mainly controlled by absorption and sedimentation of suspended solids in the estuary. Factor 3 has high positive loading of Cr and high negative loading of Se. This kind of loading of these metals indicates the variation of origin for both metals where Cr is a ferromagnesian metal mainly derived from chromite present in the Sibuti Formation (Nagarajan et al. 2017a), while Se is a chalcophile metal mainly associated with lignite, peat soils, pyrites, and/or other heavy minerals (Yudovich and Ketris 2006) in the source region. Factor 4 has higher loading of Fe and Mn observed during NEM which might be due to the formation of Fe and Mn hydroxides and co-precipitation under well-oxygenated conditions (Fig. 6d) observed during this season (Duinker et al. 1979; Wollast et al. 1979). The higher negative loading of Co and high positive loading of TSS in factor 5, and lesser representation of metals may be attributed to the adsorption by the shale concretions in Sibuti and Lambir formations under tropical conditions (Nagarajan et al. 2017a, 2015). These oxidized metals are mainly transported in dissolved form or particulate form in river systems, where both phases pose a tendency to form metal humic complexes (Tessier et al. 1984). In addition, Co is particle reactive, and 90% of Co absorption by clay minerals is common in open river streams resembling the study area, whereas desorption up to 40–70% is observed through the introduction of seawater (Anandkumar et al. 2022). Considering these observations, this association indicates the desorption of Co from suspended solids due to prevailing hydrological conditions like the flow velocity of currents, and action by waves and tides, causing the breakdown of humic complexes with the introduction of seawater in the estuary.

### Partitioning of metals between particulate and dissolved phase

**Fig. 9:**
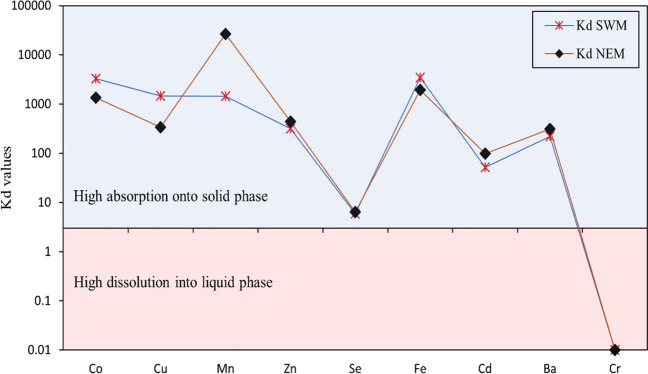
Average values of *K*_d_ for considered metals during SWM and NEM

The average partition coefficient values during both seasons for all the metals have been recorded to be greater than 3 (Fig. 9), except for Cr which is present in the liquid phase but absent in suspended solids. The higher value of *K*_d_ (< 3) shows the affinity of metals towards SS or absorption, whereas a lower value than 3 represents a higher affinity of metals towards liquid phase or dissolution under varying environmental conditions (Kumar et al. 2010; Zheng et al. 2013; Yang and Wang 2017; Zhang et al. 2018; Sedeño-Díaz et al. 2020). This infers that the metal absorption from the liquid phase and particle reactivity of metals with SS is higher, and it is a dominating process in the estuary (Li et al. 2018). The higher mobility of trace metals during SWM and NEM can be represented as Cr > Se > Cd > Ba > Zn > Mn > Cu > Co > Fe > Al and Cr > Se > Cd > Cu > Zn > Co > Ba > Fe > Mn > Al, respectively. The higher tendency of Mn to form hydroxides under oxidizing conditions because of freshwater dilution during NEM might facilitate increased reactivity towards SS (Duinker et al. 1979; Wollast et al. 1979; Oldham et al. 2017; Mori et al. 2019). On the other hand, the behaviour of Al and Cr remains the same as SWM, since the oxidizing conditions favor the formation of readily soluble Cr (VI) in water and the insoluble nature of Al under prevailing pH conditions prevents it from being mobile (Namieśnik and Rabajczyk 2010). The *K*_d_ shown by Zn, Se, Fe, and Cd is almost constant during both seasons (Fig. 9), whereas lower absorption of Co and Cu has been noticed during NEM.

### Isotopic signatures in estuarine water

The descriptive statistical composition of δD, δ^18^O, and d-excess is presented in Table 1. The composition of estuarine water samples collected during SWM and NEM shows the value of δD, ranging from − 85.41 to − 72.55‰ and − 59.06 to − 41.89‰ with respective mean values of − 79.88 and − 48.99‰. Similarly, δ^18^O in estuary deviated from − 11.20 to − 9.80‰ during SWM and − 11.02 to − 5.74‰ during NEM with mean values of − 10.67 and − 7.64‰, respectively. The value of d-excess ranged from 0.83 to 8.25‰ with a mean value of 5.47‰ during SWM, whereas it ranged from 2.89 to 29.10‰ with a mean value of 12.15‰ during NEM.

**Fig. 10:**
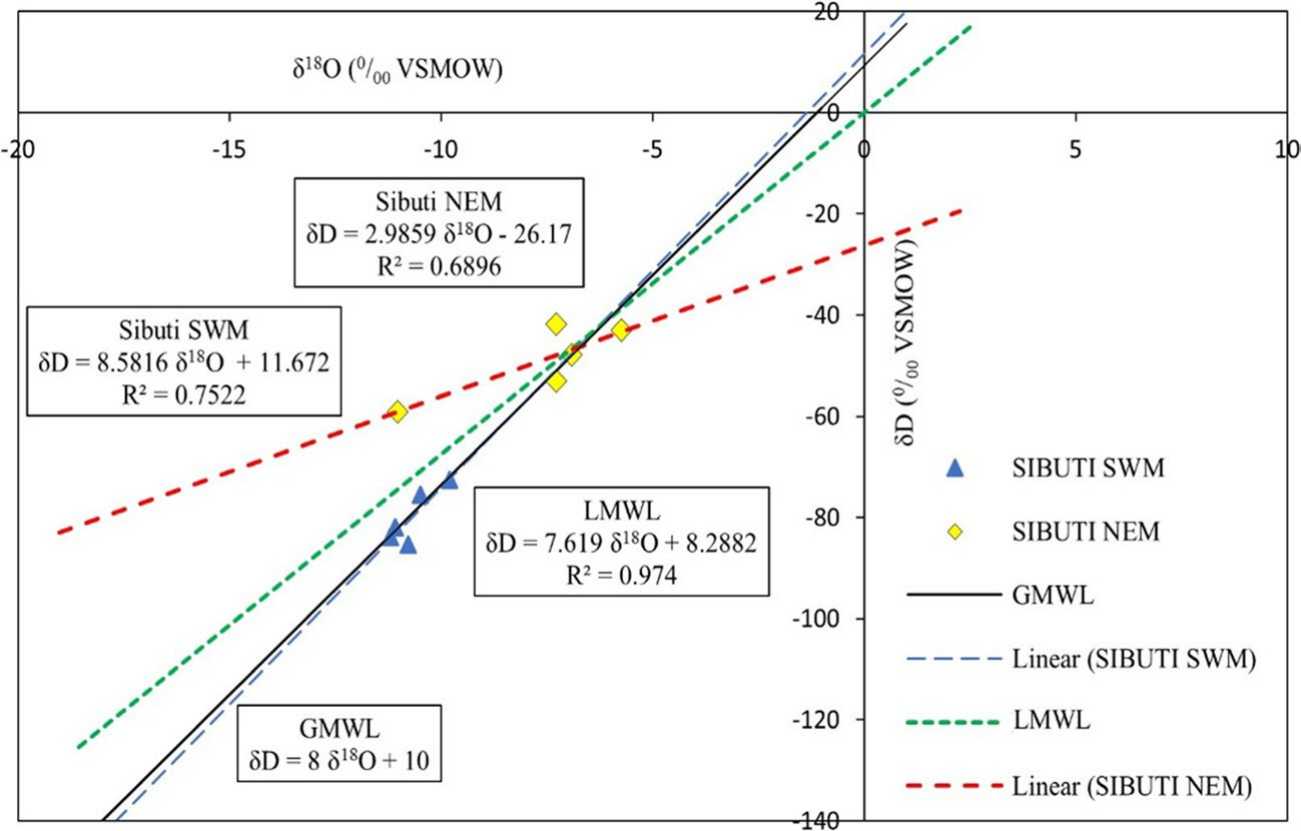
δD versus δ^18^O of Sibuti estuarine samples of SWM and NEM compared with LWML and GMWL

*X*–*Y* scatter plot was considered with fitted regression line for δ^18^O, δD, and LWML derived from Limbang area (Valappil et al. 2022), which is close to the study area (Fig. 10). The samples during SWM were plotted close to GMWL and LMWL (Fig. 10) indicating that meteoritic water was the main source of the water in the estuary, whereas NEM samples were significantly deviated from LMWL and GMWL clearly indicating an evaporation trend. However, both the regression lines intersect LWML and GMWL at an approximate common point indicating a particular location might be the source of the precipitation during both seasons (Ongetta et al. 2022). The intersection suggests that precipitation near Limbang city, Sarawak, is the main source of water in the estuary. The relationship with LMWL is determined using equations obtained in Fig. 10. The samples of SWM have a slope of 8.58 with a *d*-intercept of 11.67, whereas NEM has a slope of 2.98 with a *d*-intercept of − 26.17. This significant difference in slope indicates different moisture sources during both seasons (Datta et al. 1991). The higher slope and *d*-intercept during SWM strengthen the influence of raindrop re-evaporation (Anati and Gat 1989; Liu et al. 2014). On the other hand, a lesser slope and high negative *d*-intercept during NEM indicate intense evaporation (Liu et al. 2014).

Assessment of d-excess is useful to determine the contribution of moisture source (Gat and Carmi 1970; Deshpande et al. 2013a, b; Thivya et al. 2016; Valappil et al. 2022). Significant variable average d-excess values recorded during SWM and NEM suggest the involvement of additional processes apart from condensation from ocean evaporation or primary precipitation during the generation of moisture (Dansgaard 1964; Valappil et al. 2022; Sabarathinam et al. 2020). SWM has d-excess < 10‰, which might attribute to the secondary evaporation of raindrops due to high humidity in the source region (Clark and Fritz 1997; Gautam et al. 2017; Benetti et al. 2014). On the other hand, NEM has an average *d*-excess > 10‰, which might be due to the evaporative effect from recycled terrestrial moisture such as rivers, sea, and lakes under low humidity conditions (Chidambaram et al. 2009; Valappil et al. 2022) or a mixture of various terrestrial run-off (Deshpande et al. 2013a, b).

## Conclusion

In this study, the spatial distribution, geochemistry, and potential sources of various elements during SWM and NWM were investigated for the Sibuti River estuary and the following implications were derived:

- δD and δ^18^O values revealed that precipitation over northern Sarawak was the main source of precipitation. Though Raindrop evaporation was evident during SWM, whereas intense evaporation was noticed in NEM samples. Evaporative effluents from recycled terrestrial moisture and their re-evaporation were found to be the controlling sources for the moisture.
- Seawater dominance is consistent in the estuary irrespective of the monsoons, where Na-Cl water type is dominant during low flow, whereas mixed type Ca–Mg–Cl coupled with reverse ion exchange dominated the estuary during high flow. Similarly, tidal resistance generated by the SCS works as a key component for the dissolution of minerals where low flow is associated with increased degassing of CO_2_ due to higher residence time of water causing higher dissolution of halite and carbonates, whereas high flow with high discharge of river has a control over sulfate dissolution. The prevailing dominance of tidal water and existing hydrodynamic gradient makes the estuary more absorptive in nature by resuspension and deposition of bed load sediments as suspended solids under ideal pH conditions (4–7).
- Statistical evaluation reconfirmed the dominating tidal influence in the estuary that plays a major role in regulating the major ions (Cl^−^, Mg^2+^, Ca^2+^, Na^+^, K^+^, HCO_3_^−^, and SO_4_^2−^) irrespective of the seasons. The nitrogen cycle is evident in the estuary, where nitrification, denitrification, and ammonification are major processes during both seasons in the lower and intermediate zones of the estuary. In addition, the oxidation of organic matter was dominant in the turbidity maximum zone (TMZ) due to anaerobic conditions prevailing near the river mouth giving rise to organic-induced negatively charged Mn-oxyhydroxide collides, control the absorption in the lower reaches during both seasons facilitating Cd and Zn concentrations in suspended solids. The absorption in the intermediate zone and upper reaches are controlled by Al-oxyhydroxides and Fe-oxyhydroxides, and their settlement in the lower reaches due to increased seawater influence makes them dominant in the sediments and thus serve as major carriers of metals such as Co, Cr, Ba, Se, Cu, and Pb.
- Nutrients such as NO_3_^−^, NH_3_, NH_4_^+^, and PO_4_^3−^ were originated from agricultural activities in the river basin whereas wastewater effluents from Bekenu were found to be the major contributor of NH_3_. The trace metals mainly originated from geogenic sources, especially from shale and pyrite concretion of the Sibuti Formation and siliciclastic sediments of the Sibuti and Lambir formations. The study also infers that oxidation and high deforestation coupled with a high degree of chemical weathering due to higher rainfall might be the major contributor for the higher concentrations of trace metals in the river and estuary.

## Supplementary Information

Below is the link to the electronic supplementary material.

Supplementary file1 (DOCX 1518 KB)

Supplementary file2 (XLSX 46 KB)

## Data Availability

The datasets used and analyzed during the current study are available from the corresponding author on reasonable request.
