# Supplementary material for: Spatial and temporal variations of geochemical processes and toxicity of water, sediments, and suspended solids in Sibuti River Estuary, NW Borneo
Source: Environ Sci Pollut Res Int. 2023 Jul 26;30(40):92692–719. doi: 10.1007/s11356-023-28596-5 (PMC10447316; doi:10.1007/s11356-023-28596-5)
Supplement: Supplementary file 1 — Supplementary file1 (DOCX 1518 KB) [file 11356_2023_28596_MOESM1_ESM.docx]

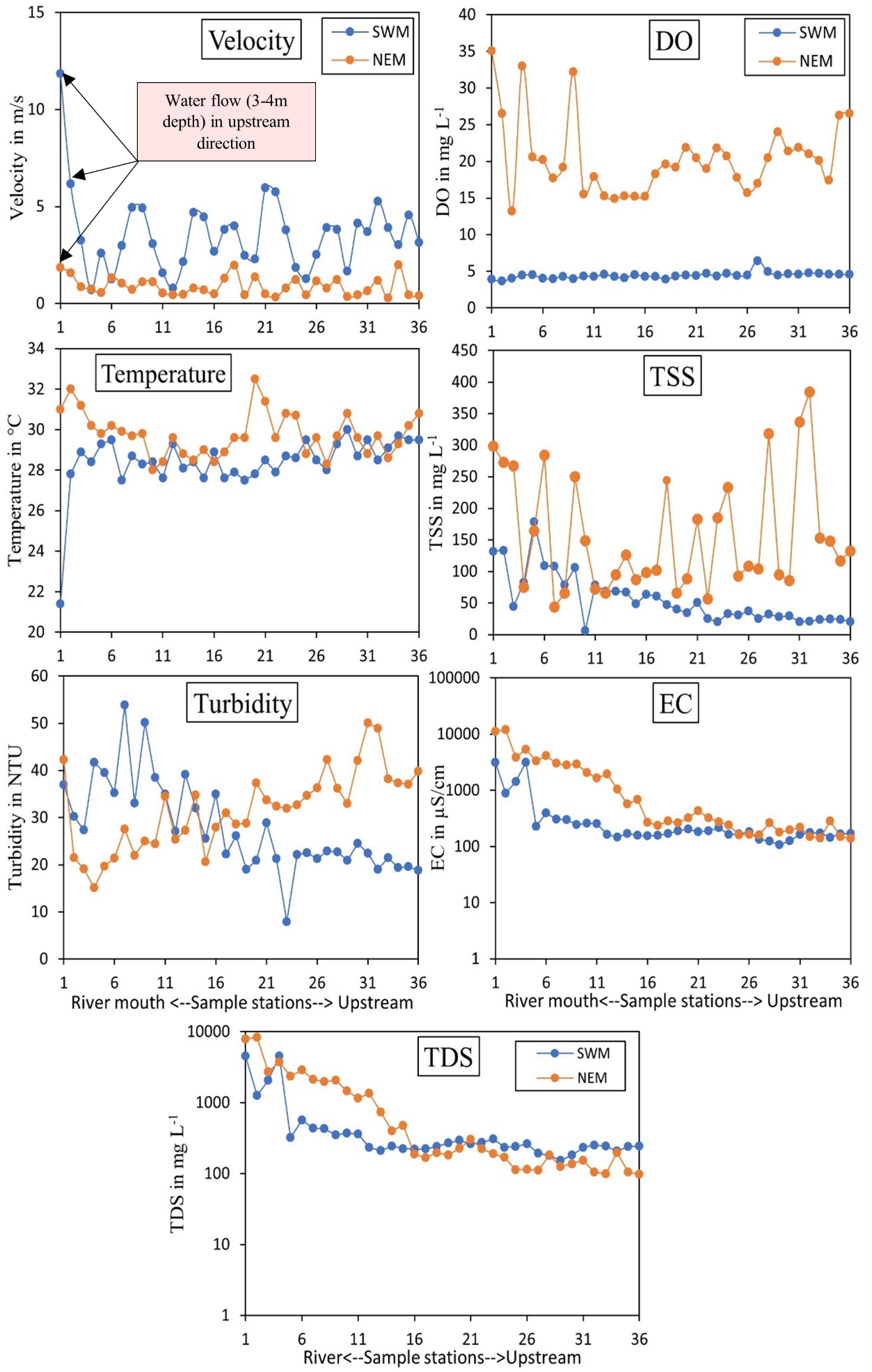
***Supplementary Fig. 1 Variation of Turbidity, Velocity, DO, Temperature and TDS during SWM and NEM***

***Supplementary Fig. 2 Comparison of Salinity with tidal influence in the river during SWM and NEM***


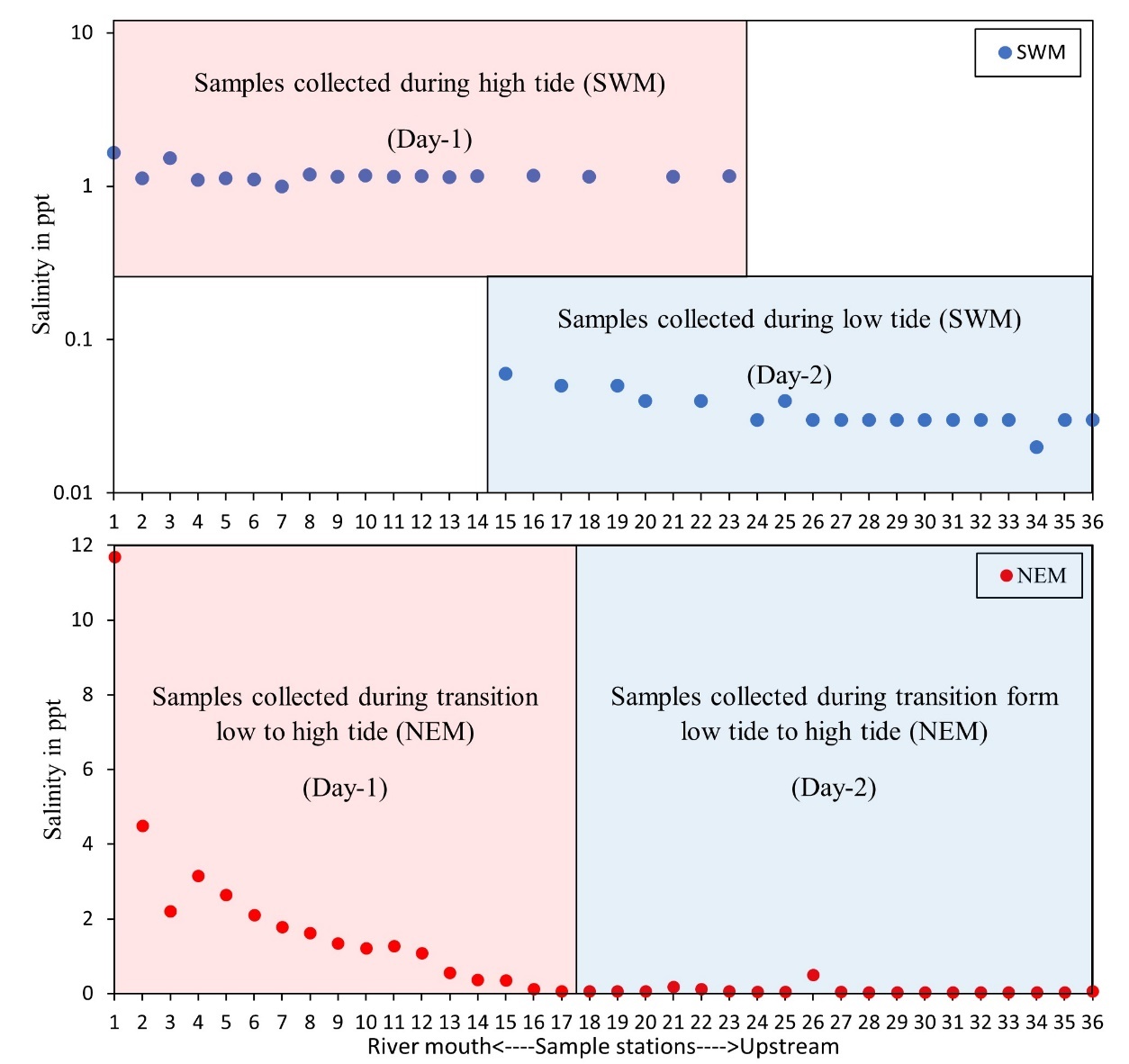


***Supplementary Table 1 Instrument conditions of AAS for various elements as per Perkins Elmar recommendations.***

| **Element** | **Wavelength (nm)** | **Char. Conc (mg/l) ^a^** | **Sensitivity Check (mg/l) ^b^** | **Linear Range (mg/l) ^c^** | **Slit Width**  **(nm)** | **Oxidant** | **Oxidant Flow (L/min)** | **Acetylene/N_2_O Flow (L/min)** | **Light Sources (Lamp) ^d^** |
| --- | --- | --- | --- | --- | --- | --- | --- | --- | --- |
| **Co** | 243.58 | 0.4 | 20 | - | 1.8/1.35 | Air | 10 | 2.5 | HCL |
| **Cd** | 228.80 | 0.01 | 0.5 | 1 | 2.7/1.35 | Air | 10 | 2.5 | EDL & HCL |
| **Cu** | 216.51 | 0.15 | 6.5 | 6.5 | 1.8/1.35 | Air | 10 | 2.5 | HCL |
| **Fe** | 346.59 | 3.6 | 180 | - | 1.8/0.6 | Air | 10 | 2.5 | HCL |
| **Pb** | 283.31 | 0.18 | 8 | 10 | 2.7/1.05 | Air | 10 | 2.5 | EDLs & HCL |
| **Mn** | 279.83 | 0.016 | 1 | 0.6 | 1.8/0.6 | Air | 10 | 2.5 | HCL |
| **Zn** | 213.86 | 0.006 | 0.3 | 0.75 | 2.7/1.8 | Air | 10 | 2.5 | HCL |
| **Se** | 196.03 | 0.3 | 15 | 100 | 2.7/2.3 | Air | 10 | 2.5 | HCL |
| **Al** | 309.27 | 1.1 | 50 | 100 | 2.7/0.8 | N_2_O | 6 | 7.5 | HCL |
| **Cr** | 357.87 | 0.078 | 4 | 5 | 2.7/0.8 | N_2_O | 10 | 3.3 | HCL |
| **Ba** | 553.55 | 0.46 | 20 | 20 | 1.8/0.6 | N_2_O | 6.2 | 8.2 | HCL |
| **Na^+^** | 330.24 | 0.9 | 45 | - | 2.1/0.8 | Air | 10 | 2.5 | HCL |
| **K^+^** | 404.41 | 4 | 180 | 300 | 2.7/0.6 | Air | 10 | 2.5 | HCL |

^a^ Characteristic Concentration is the minimum absorbance value of the instrument.

^b^ Sensitivity check is the concentration giving approximately 0.2 absorbance unit (AU)

^c^ Linear range is the maximum absorbance value of the instrument

^d^ Light source is the Electrode Cathode Lamps (EDL) and Hollow Cathode Lamp (HCL)
